# Supplementary material for: Defining the RBPome of primary T helper cells to elucidate higher-order Roquin-mediated mRNA regulation
Source: Nat Commun. 2021 Sep 1;12:5208. doi: 10.1038/s41467-021-25345-5 (PMC8410761; doi:10.1038/s41467-021-25345-5)
Supplement: Supplementary file 1 — Supplementary Information [file 41467_2021_25345_MOESM1_ESM.pdf]

Defining the RBPome of primary T helper cells to elucidate higher-order Roquin-mediated mRNA regulation

Hoefig et al.

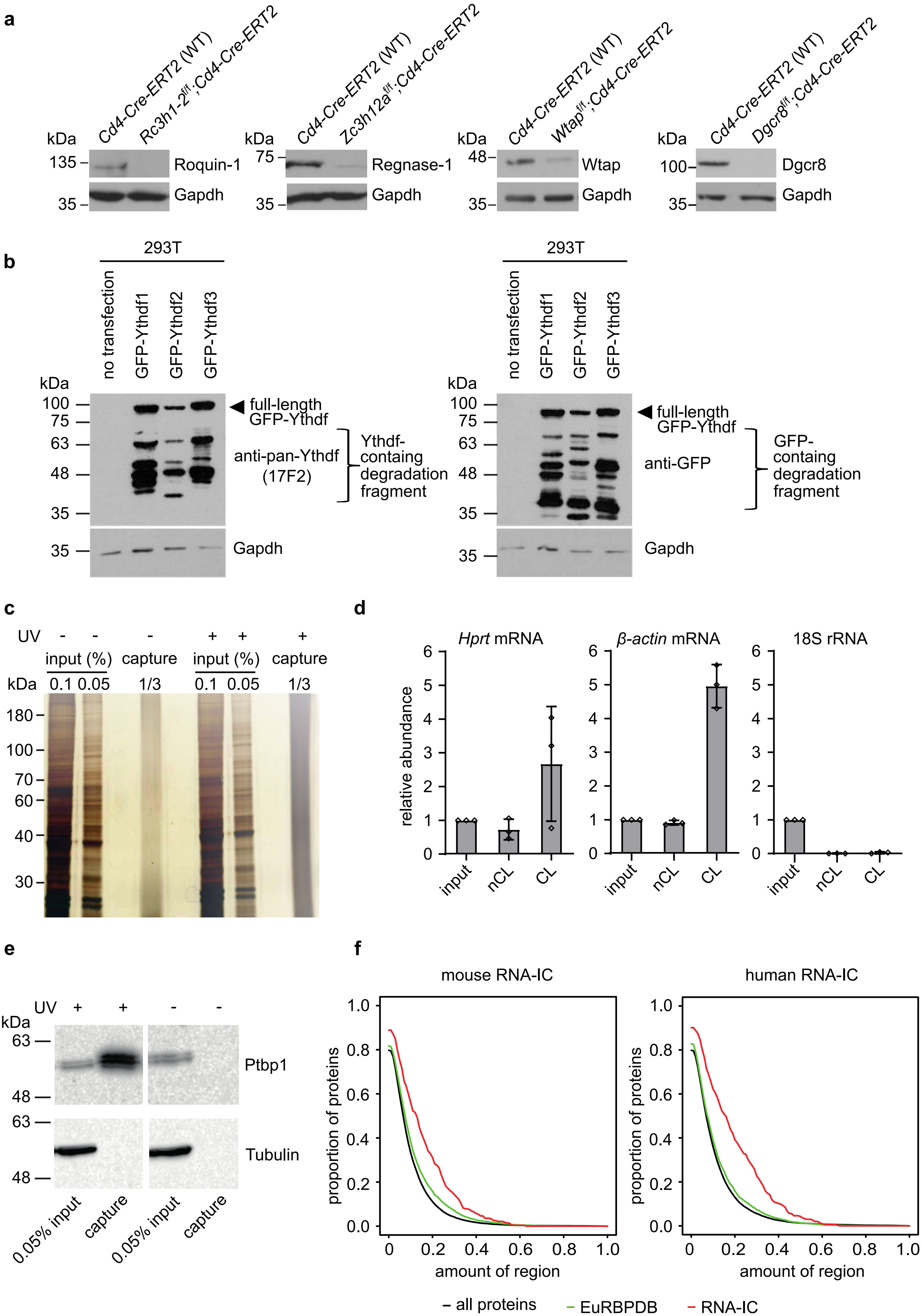
a
Cd4-Cre-ERT2 (WT)
Rc3h1-2f/f;Cd4-Cre-ERT2
kDa
135
35
Roquin-1
Gapdh
Zc3h12af/f;Cd4-Cre-ERT2
75
Regnase-1
Wtapf/f;Cd4-Cre-ERT2
48
Wtap
Dgcr8f/f;Cd4-Cre-ERT2
100
Dgcr8
b
293T
no transfection
GFP-Ythdf1
GFP-Ythdf2
GFP-Ythdf3
full-length GFP-Ythdf
anti-pan-Ythdf (17F2)
Ythdf-containg degradation fragment
anti-GFP
GFP-containg degradation fragment
c
UV
input (%)
capture
0.1 0.05
1/3
180
100
70
60
40
30
d
Hprt mRNA
β-actin mRNA
18S rRNA
relative abundance
input
nCL
CL
e
UV
Ptbp1
Tubulin
0.05% input
capture
63
48
f
mouse RNA-IC
human RNA-IC
proportion of proteins
amount of region
all proteins
EuRBPDB
RNA-IC

Suppl. Fig. 1

Supplementary Fig. 1: RNA-IC supporting results. (**a**) Western blots showing depletion of Roquin-1/2, Regnase-1, Wtap and Dgcr8 on day 5 after $CD4^+$ T cell activation (n=1), supporting FACS data from **Fig. 1 c, f, i** and **l**. (**b**) Western blots demonstrating specificity of the newly established pan-Ythdf monoclonal antibody 17F2 for N-terminal GFP fusions of Ythdf1, Ythdf2 and Ythdf3, with no signals being present in non-transfected 293T cells (n=1). (**c**) Silver staining analysis of oligo(dT)-captured samples with and without UV irradiation (n=2). (**d**) Quantitative RT-PCR to determine RNA pull-down efficiency with (CL) and without crosslink (nCL). Error bars indicate the standard deviation and the mean derived from three independent experiments. (**e**) Western blotting of UV irradiated and nonirradiated samples of EL-4 T cells. Membranes were probed with antibodies for the known RNA-binding protein polypyrimidine tract binding protein 1 (Ptbp1) and β-tubulin (n=2). (**f**) Distribution of low complexity regions in all Uniprot reviewed protein sequences (black line), in proteins in the EuRBPDB database (green) and in proteins significant in the RNA-IC data (red line). The left plot shows mouse data and the right plot human data. According to two-sided Kolmogorov-Smirnov testing the LCR distribution differences between RNA-IC (red lines) and all proteins (black lines) are highly significant in mouse ($p<2.2x10^{-16}$) and man ($p=7.8x10^{-16}$). Source data are provided as a Source Data file.

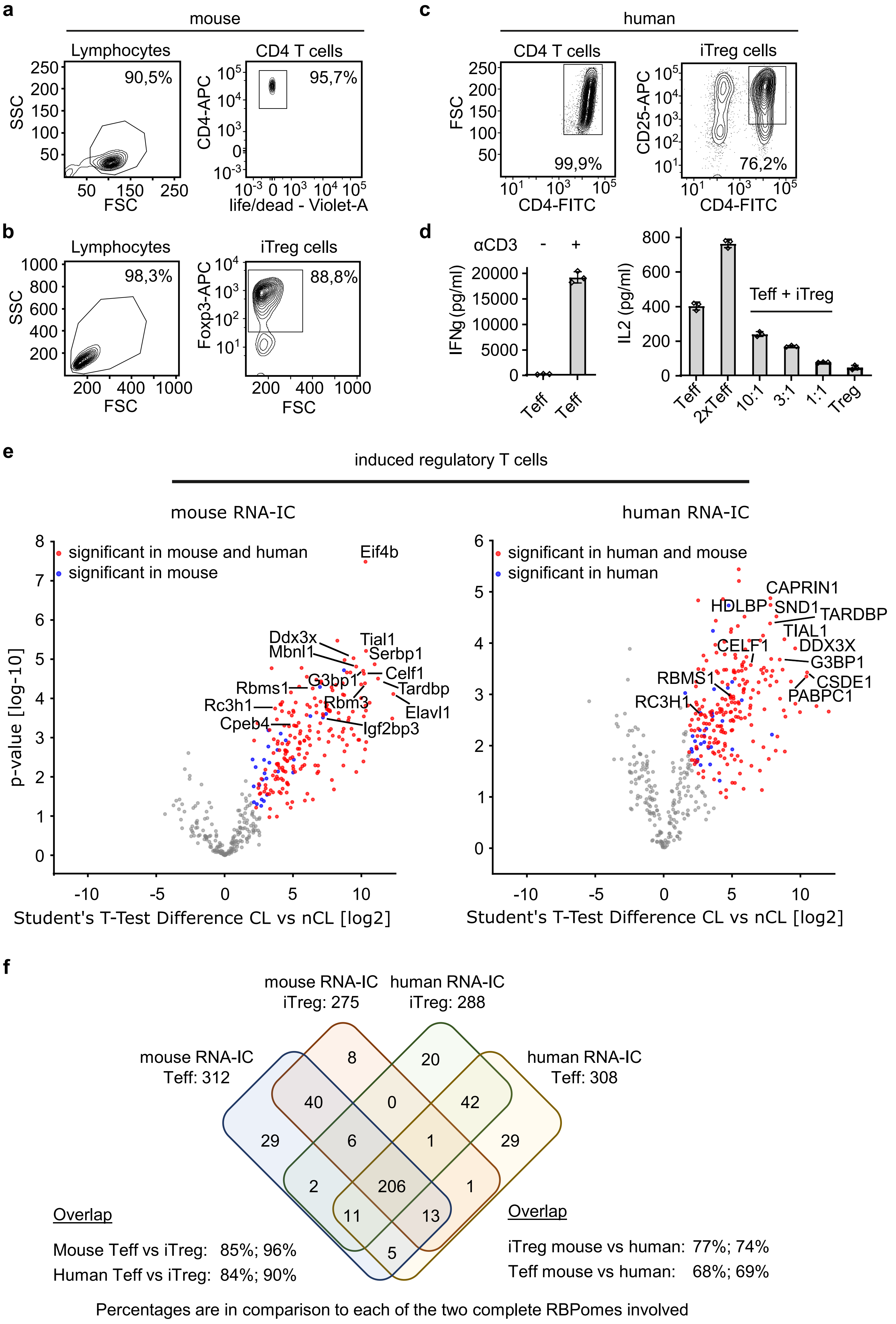
a
mouse
Lymphocytes
90,5%
SSC
FSC
CD4 T cells
95,7%
CD4-APC
life/dead - Violet-A
b
Lymphocytes
98,3%
SSC
FSC
iTreg cells
88,8%
Foxp3-APC
FSC
c
human
CD4 T cells
FSC
99,9%
CD4-FITC
iTreg cells
CD25-APC
76,2%
CD4-FITC
d
αCD3
-
+
IFNg (pg/ml)
Teff
Teff
IL2 (pg/ml)
Teff + iTreg
Teff
2xTeff
10:1
3:1
1:1
Treg
e
induced regulatory T cells
mouse RNA-IC
significant in mouse and human
significant in mouse
Eif4b
Ddx3x
Mbnl1
Tial1
Serbp1
G3bp1
Celf1
Rbms1
Tardbp
Rbm3
Rc3h1
Elavl1
Cpeb4
Igf2bp3
p-value [log-10]
Student's T-Test Difference CL vs nCL [log2]
human RNA-IC
significant in human and mouse
significant in human
CAPRIN1
HDLBP
SND1
TARDBP
TIAL1
CELF1
DDX3X
G3BP1
RBMS1
CSDE1
PABPC1
RC3H1
Student's T-Test Difference CL vs nCL [log2]
f
mouse RNA-IC
iTreg: 275
human RNA-IC
iTreg: 288
mouse RNA-IC
Teff: 312
human RNA-IC
Teff: 308
8
20
40
0
42
29
6
1
29
2
206
1
11
13
5
Overlap
Mouse Teff vs iTreg: 85%; 96%
Human Teff vs iTreg: 84%; 90%
Overlap
iTreg mouse vs human: 77%; 74%
Teff mouse vs human: 68%; 69%
Percentages are in comparison to each of the two complete RBPomes involved

Suppl. Fig. 2

Supplementary Fig. 2: RNA-IC on mouse and human iTreg cells. (**a, b**) Exemplarily shown are kit-purified ‘untouched’ mouse $CD4^+$ T cells that are alive, of high purity (**a**) and can routinely be skewed towards the iTreg subset with high efficiency (**b**). (**c, d**) Functional human $CD4^+$ T cells (**c**, left panel) have been used for RNA-IC (n=3 donors). Left-over resting Teff cells (day 19) stayed untreated or were challenged with 1.5 µg/ml of the αCD3 antibody OKT3 to produce the T_H1 signature cytokine IFNγ (**d**, left panel), which was measured by ELISA in triplicates (n=3). Error bars, mean ± s.d.. Functional human iTreg cells from three donors have been used for RNA-IC, as their presence could reduce IL-2 secretion (**c, d**, right panels) by the EBV-specific $CD4^+$ T cell clone BALF4-B5 (Teff) upon recognition of cognate antigen on autologous APC. IL-2 in the supernatant was measured after 24h by ELISA in triplicates (n=3). Error bars, mean ± s.d.. (**e**) Volcano plots from two-sided Student's T-test analysis using a permutation-based FDR method for multiple hypothesis corrections showing the $-\log_{10}$ p-value in relation to the $\log_2$ fold-change comparing the RNA-capture from crosslinked mouse induced regulatory T cells (left plot) or human induced regulatory T cells (right plot) in comparison to the non-crosslinked control. Red dots represent proteins significant at a 5% FDR cutoff level in both mouse and human RNA-capture experiments and blue dots proteins were significant only in mouse or human, respectively. (**f**) Venn diagram using four datasets to compare RNA-IC derived RBPomes of effector T and induced Treg cells from mouse and man. Source data are provided as a Source Data file.

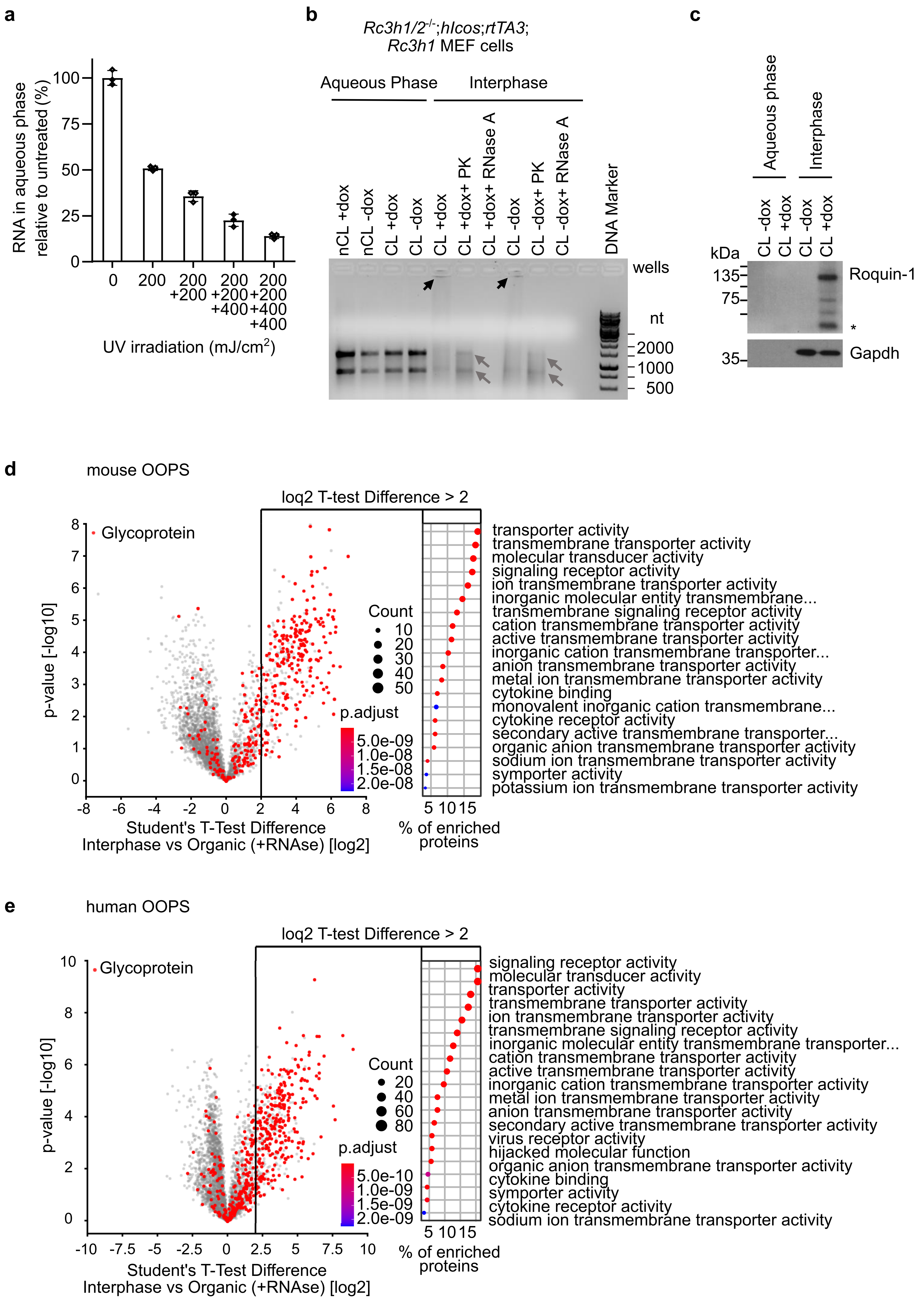

a
RNA in aqueous phase relative to untreated (%)
100
75
50
25
0
0
200
200 +200
200 +200 +400
200 +200 +400 +400
UV irradiation (mJ/cm2)
b
Rc3h1/2-/-;hIcos;rtTA3; Rc3h1 MEF cells
Aqueous Phase
Interphase
nCL +dox
nCL -dox
CL +dox
CL -dox
CL +dox
CL +dox+ PK
CL +dox+ RNase A
CL -dox
CL -dox+ PK
CL -dox+ RNase A
DNA Marker
wells
nt
2000
1000
500
c
Aqueous phase
Interphase
CL -dox
CL +dox
CL -dox
CL +dox
kDa
135
75
35
Roquin-1
*
Gapdh
d
mouse OOPS
loq2 T-test Difference > 2
Glycoprotein
p-value [-log10]
Count
10
20
30
40
50
p.adjust
5.0e-09
1.0e-08
1.5e-08
2.0e-08
Student's T-Test Difference
Interphase vs Organic (+RNAse) [log2]
% of enriched proteins
transporter activity
transmembrane transporter activity
molecular transducer activity
signaling receptor activity
ion transmembrane transporter activity
inorganic molecular entity transmembrane...
transmembrane signaling receptor activity
cation transmembrane transporter activity
active transmembrane transporter activity
inorganic cation transmembrane transporter...
anion transmembrane transporter activity
metal ion transmembrane transporter activity
cytokine binding
monovalent inorganic cation transmembrane...
cytokine receptor activity
secondary active transmembrane transporter...
organic anion transmembrane transporter activity
sodium ion transmembrane transporter activity
symporter activity
potassium ion transmembrane transporter activity
e
human OOPS
loq2 T-test Difference > 2
Glycoprotein
p-value [-log10]
Count
20
40
60
80
p.adjust
5.0e-10
1.0e-09
1.5e-09
2.0e-09
Student's T-Test Difference
Interphase vs Organic (+RNAse) [log2]
% of enriched proteins
signaling receptor activity
molecular transducer activity
transporter activity
transmembrane transporter activity
ion transmembrane transporter activity
transmembrane signaling receptor activity
inorganic molecular entity transmembrane transporter...
cation transmembrane transporter activity
active transmembrane transporter activity
inorganic cation transmembrane transporter activity
metal ion transmembrane transporter activity
anion transmembrane transporter activity
secondary active transmembrane transporter activity
virus receptor activity
hijacked molecular function
organic anion transmembrane transporter activity
cytokine binding
symporter activity
cytokine receptor activity
sodium ion transmembrane transporter activity

Suppl. Fig. 3

Supplementary Fig. 3: OOPS supporting results. (**a**) To meet published recommendations for performing OOPS experiments, CD4$^+$ T cells were treated with increasing doses of UV irradiation to determine at which point 75% of the RNA was engaged in crosslinks with proteins and hence was depleted from the aqueous phase. T cells were subjected to individual UV doses of 200 or 400 mJ per cm^2, as indicated, while floating in 6well dishes on icy water and with intermittent shaking. After phase partitioning, the aqueous phase from each sample was split into three prior to RNA isolation to account for precipitation deviations (n=1). Error bars of three technical replicates, mean ± s.d.. (**b**) Agarose gel demonstrating disappearance of the typical 18S/28S rRNA bands after crosslinking and appearance of upshifted protein-RNA adducts (black arrows) in MEF cells with and without doxycycline-induce Roquin-1 expression. rRNA bands reappear after proteinase K (PK) treatment (grey arrows) and after RNase treatment the protein-RNA adducts in the wells disappear (n=2). (**c**) Western blots showing that known RBPs, such as Roquin-1 and Gapdh can be detected in interphases, in the case of Roquin-1 only after induced expression. * cleavage product (n=1). (**d, e**) Volcano plot from two-sided Student's T-test analysis using a permutation-based FDR method for multiple hypothesis corrections showing the $-\log_{10}$ p-value plotted against the $\log_2$ fold-change comparing the interphase of OOPS experiments of crosslinked mouse (**d**) or human (**e**) CD4$^+$ T cells versus the organic phase after RNase treatment of the interphase. Glycoproteins are highlighted in red. Enrichment analysis of GO Molecular Function terms was performed for proteins with a $\log_2$ fold-change larger than 2. The 20 most enriched terms are depicted. Source data are provided as a Source Data file.

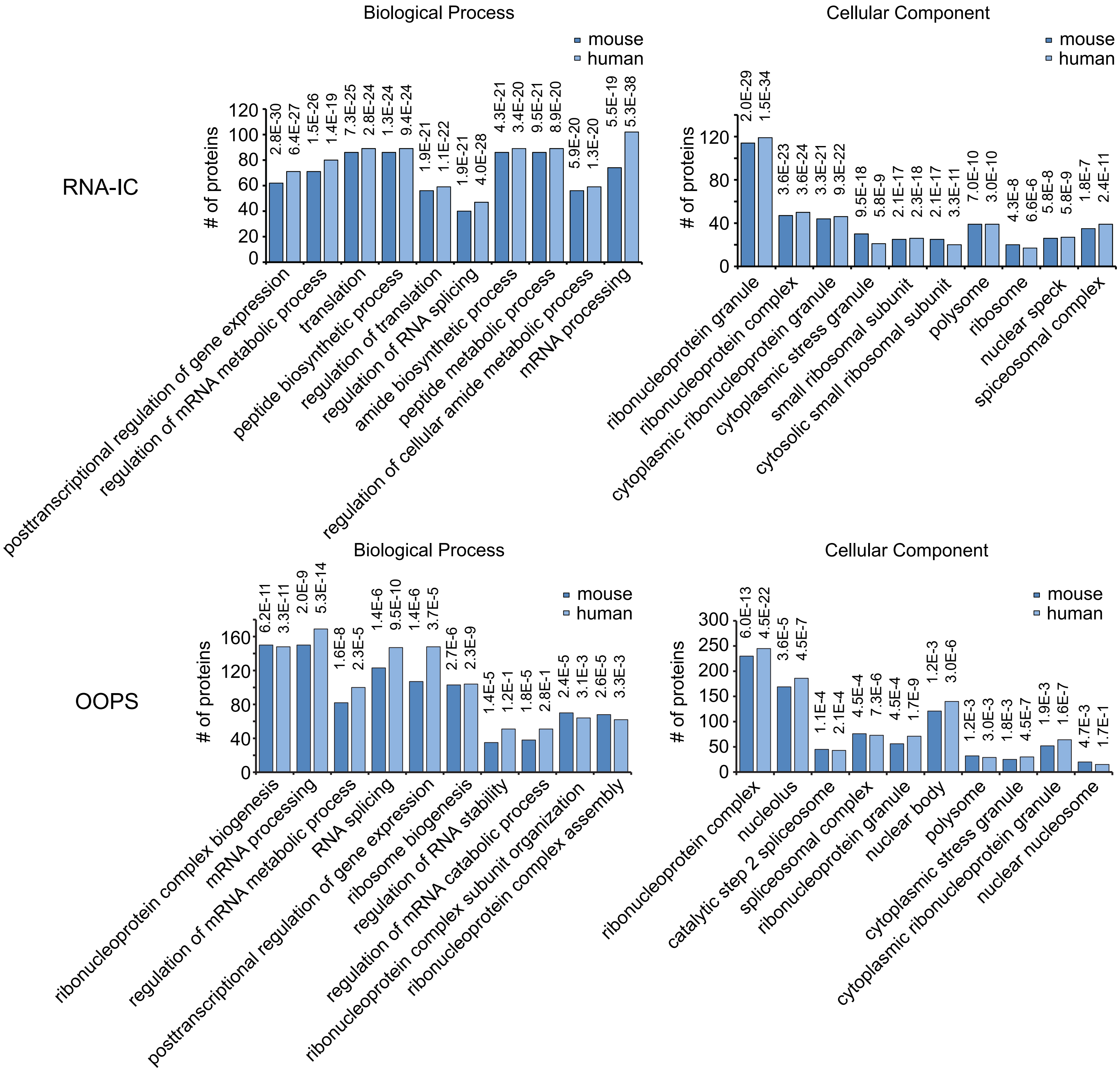

RNA-IC
Biological Process
mouse
human
# of proteins
0
20
40
60
80
100
120
2.8E-30
6.4E-27
1.5E-26
1.4E-19
7.3E-25
2.8E-24
1.3E-24
9.4E-24
1.9E-21
1.1E-22
1.9E-21
4.0E-28
4.3E-21
3.4E-20
9.5E-21
8.9E-20
5.9E-20
1.3E-20
5.5E-19
5.3E-38
posttranscriptional regulation of gene expression
regulation of mRNA metabolic process
translation
peptide biosynthetic process
regulation of translation
regulation of RNA splicing
amide biosynthetic process
peptide metabolic process
regulation of cellular amide metabolic process
mRNA processing
Cellular Component
mouse
human
# of proteins
0
40
80
120
2.0E-29
1.5E-34
3.6E-23
3.6E-24
3.3E-21
9.3E-22
9.5E-18
5.8E-9
2.1E-17
2.3E-18
2.1E-17
3.3E-11
7.0E-10
3.0E-10
4.3E-8
6.6E-6
5.8E-8
5.8E-9
1.8E-7
2.4E-11
ribonucleoprotein granule
ribonucleoprotein complex
cytoplasmic ribonucleoprotein granule
cytoplasmic stress granule
small ribosomal subunit
cytosolic small ribosomal subunit
polysome
ribosome
nuclear speck
spiceosomal complex
OOPS
Biological Process
mouse
human
# of proteins
0
40
80
120
160
6.2E-11
3.3E-11
2.0E-9
5.3E-14
1.6E-8
2.3E-5
1.4E-6
9.5E-10
1.4E-6
3.7E-5
2.7E-6
2.3E-9
1.4E-5
1.2E-1
1.8E-5
2.8E-1
2.4E-5
3.1E-3
2.6E-5
3.3E-3
ribonucleoprotein complex biogenesis
mRNA processing
regulation of mRNA metabolic process
RNA splicing
posttranscriptional regulation of gene expression
ribosome biogenesis
regulation of RNA stability
regulation of mRNA catabolic process
ribonucleoprotein complex subunit organization
ribonucleoprotein complex assembly
Cellular Component
mouse
human
# of proteins
0
50
100
150
200
250
300
6.0E-13
4.5E-22
3.6E-5
4.5E-7
1.1E-4
2.1E-4
4.5E-4
7.3E-6
4.5E-4
1.7E-9
1.2E-3
3.0E-6
1.2E-3
3.0E-3
1.8E-3
4.5E-7
1.9E-3
1.6E-7
4.7E-3
1.7E-1
ribonucleoprotein complex
nucleolus
catalytic step 2 spliceosome
spliceosomal complex
ribonucleoprotein granule
nuclear body
polysome
cytoplasmic stress granule
cytoplasmic ribonucleoprotein granule
nuclear nucleosome

Suppl. Fig. 4

Supplementary Figure 4: Gene ontology enrichment analysis. Enrichment analysis of GO Biological Process and GO Cellular Component terms of significant proteins in mouse or human RNA-IC data (top row) or OOPS data (bottom row). The ten most enriched terms in mouse (dark blue) and the respective terms in human (light blue) are shown. The y-axis represents the number of proteins matching the respective GO term. P-values were calculated using the hypergeometric distribution and were adjusted by Benjamini-Hochberg multiple testing correction. Numbers above each term depict the adjusted p-value.

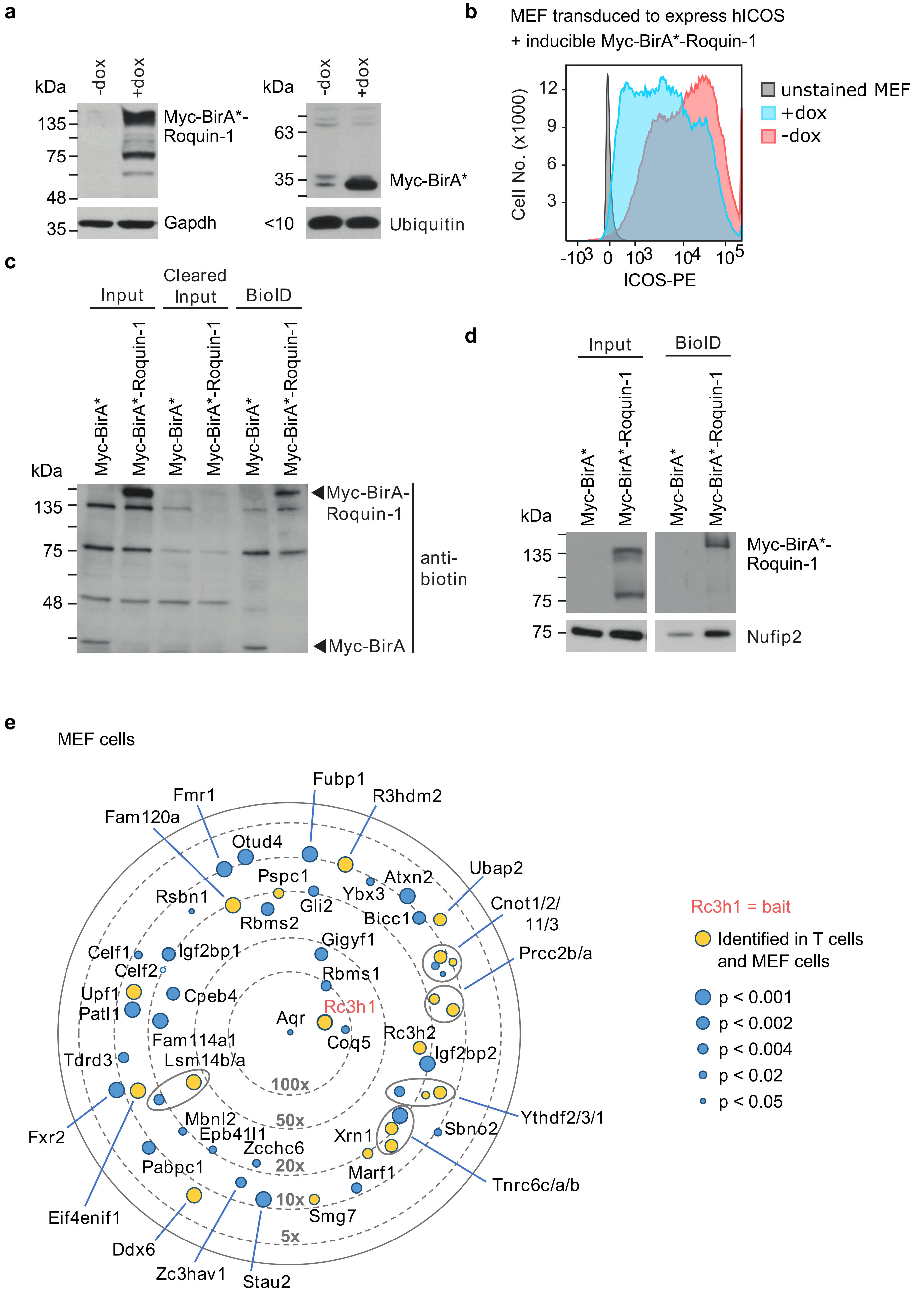
a
kDa
-dox
+dox
135
75
48
35
Myc-BirA*-Roquin-1
Gapdh
63
35
<10
Myc-BirA*
Ubiquitin
b
MEF transduced to express hICOS
+ inducible Myc-BirA*-Roquin-1
Cell No. (x1000)
12
9
6
3
-10^3 0 10^3 10^4 10^5
ICOS-PE
unstained MEF
+dox
-dox
c
Input
Cleared Input
BioID
Myc-BirA*
Myc-BirA*-Roquin-1
kDa
135
75
48
Myc-BirA-Roquin-1
anti-biotin
Myc-BirA
d
Input
BioID
Myc-BirA*
Myc-BirA*-Roquin-1
kDa
135
75
Myc-BirA*-Roquin-1
Nufip2
e
MEF cells
Fubp1
Fmr1
R3hdm2
Fam120a
Otud4
Pspc1
Atxn2
Ubap2
Rsbn1
Ybx3
Gli2
Rbms2
Bicc1
Cnot1/2/11/3
Celf1
Igf2bp1
Gigyf1
Prcc2b/a
Celf2
Rbms1
Upf1
Cpeb4
Patl1
Rc3h1
Aqr
Coq5
Rc3h2
Fam114a1
Igf2bp2
Tdrd3
Lsm14b/a
100x
50x
Ythdf2/3/1
Mbnl2
Epb41l1
Sbno2
Fxr2
Xrn1
Zcchc6
Pabpc1
20x
Marf1
Tnrc6c/a/b
10x
Eif4enif1
Smg7
5x
Ddx6
Zc3hav1
Stau2
Rc3h1 = bait
Identified in T cells and MEF cells
p < 0.001
p < 0.002
p < 0.004
p < 0.02
p < 0.05

Suppl. Fig. 5

Supplementary Fig. 5: Identification of Roquin-1 preys by BioID in MEF cells. (**a**) Western blots showing doxycycline-induced expression of Myc-BirA*-Roquin-1 or Myc-BirA* in MEF cell clones. (**b**) FACS blot demonstrating that as in T cells (**Fig. 6c**) N-terminal fusion of Myc-BirA* to Roquin-1 does not affect its function and (**c, d**) the fusion protein can biotinylate Roquin-1 and (**d**) its cofactor Nufip2. (**e**) Identified preys from Roquin-1 BioID in MEF cell clones. Depicted are 55 of 143 significantly enriched proteins (n=4) for better comparison with **Fig. 6d**. Yellow dot color indicates identification of the Roquin-1 prey in MEF and T cells. Dot sizes equal p-values and positioning towards the center implies increased x-fold enrichment. A two-sided heteroscedastic Student's T-test analysis was performed. Source data are provided as a Source Data file.

a

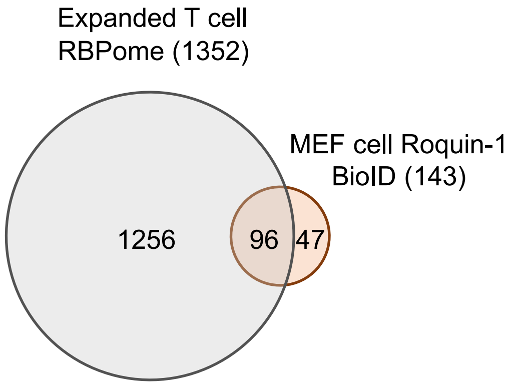

b

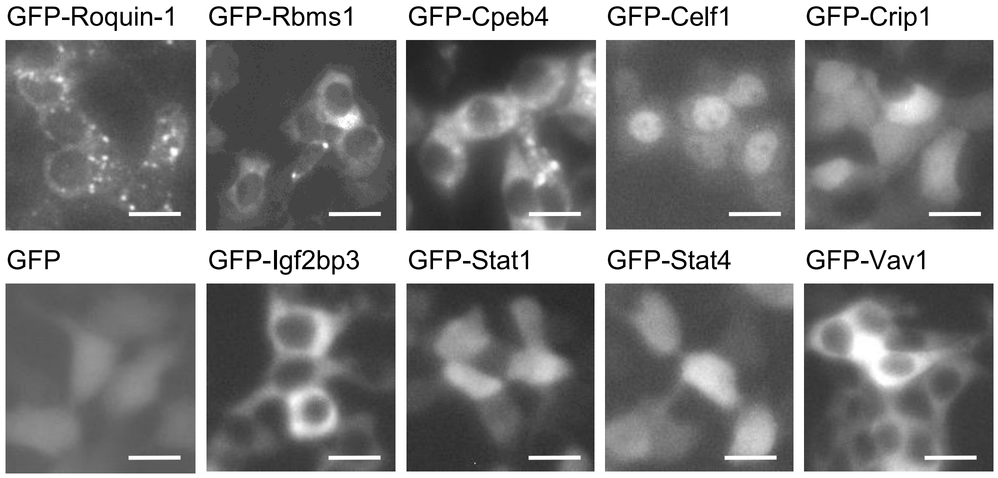

Suppl. Fig. 6

Supplementary Fig. 6: Overlap between Roquin-1 preys in MEF cells and the CD4$^+$ T cell RBPome. (**a**) Venn diagram showing that 96 proteins (67%) are Roquin-1 preys and also RBPs in T cells. (**b**) Microscopical images showing different localizations of the GFP signals as a result of GFP-GOI expression in transfected 293T cells. Scale bars represent 20 µm.

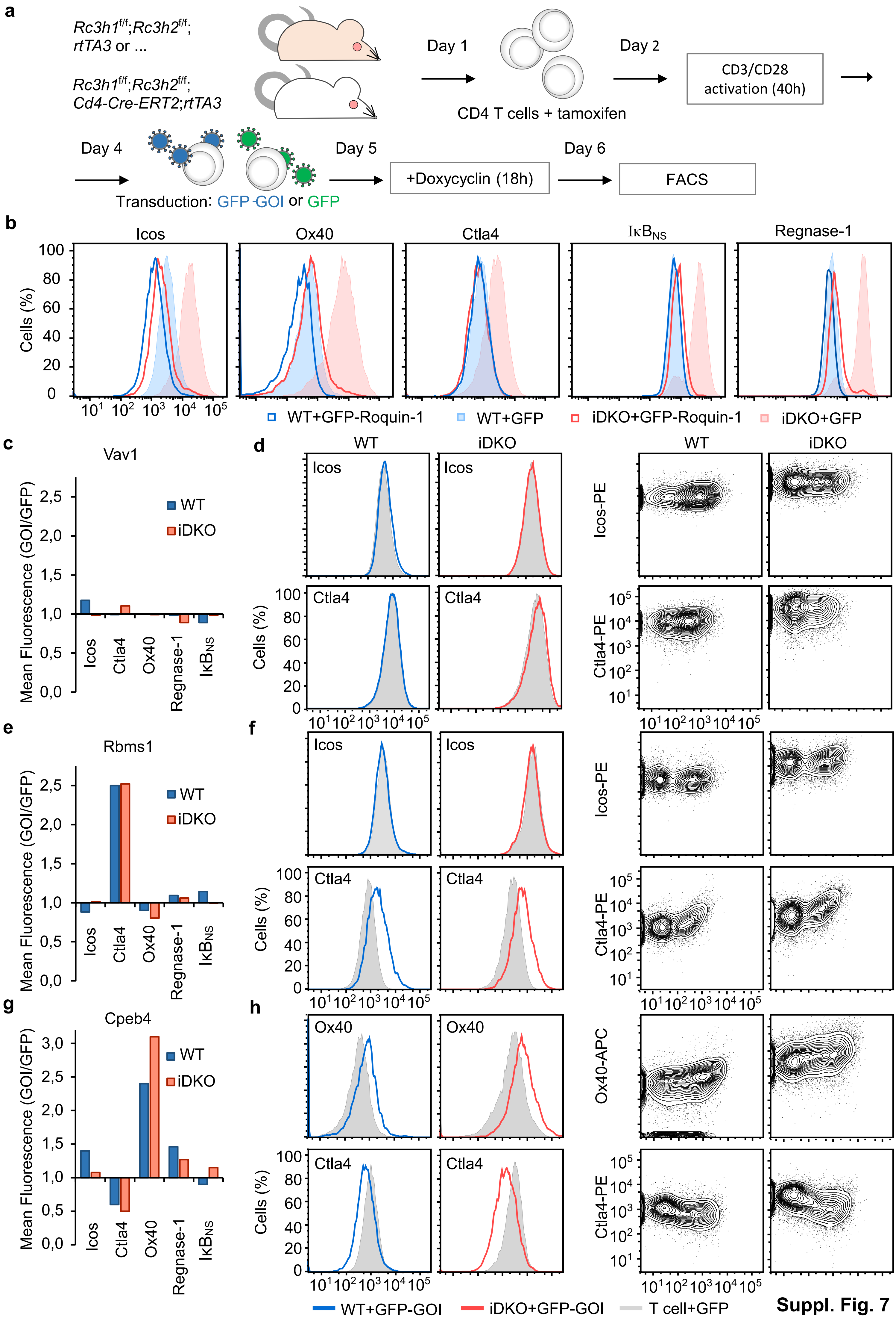

a
Rc3h1f/f;Rc3h2f/f; rtTA3 or ...
Rc3h1f/f;Rc3h2f/f; Cd4-Cre-ERT2;rtTA3
Day 1
CD4 T cells + tamoxifen
Day 2
CD3/CD28 activation (40h)
Day 4
Transduction: GFP-GOI or GFP
Day 5
+Doxycyclin (18h)
Day 6
FACS
b
Icos
Ox40
Ctla4
IκBNS
Regnase-1
Cells (%)
WT+GFP-Roquin-1
WT+GFP
iDKO+GFP-Roquin-1
iDKO+GFP
c
Vav1
Mean Fluorescence (GOI/GFP)
WT
iDKO
Icos
Ctla4
Ox40
Regnase-1
IκBNS
d
WT
iDKO
Icos
Ctla4
Cells (%)
Icos-PE
Ctla4-PE
e
Rbms1
f
g
Cpeb4
h
Ox40
Ox40-APC
WT+GFP-GOI
iDKO+GFP-GOI
T cell+GFP

Suppl. Fig. 7

Supplementary Fig. 7: Supporting results for higher order Icos regulation by Roquin-1 and Celf1 or Igf2bp3. (**a**) Schematic representation of the experiment performed in **Fig. 7**. (**b**) Treatment with 4'-OH-tamoxifen of $CD4^+$ T cells with the genotypes *Rc3h1*$^{f/f}$;*Rc3h2*$^{f/f}$;*rtTA3* without (WT) or with the *Cd4-Cre-ERT2* allele (iDKO) were used for transduction with a retrovirus expressing GFP-Roquin-1. Expression levels of the Roquin-1 targets Icos, Ox40, Ctla4 and $Iκb_{NS}$ were analyzed and work as a positive control for the experiment. (**c, e, g**) The geometrical mean of each GOI-GFP divided by GFP for each Roquin-1 target was calculated and the summarized results for one negative example (Vav1) and two Roquin-1-independend regulations (Rbms1 and Cpeb4) are shown as bar diagrams. (**d, f, h**) Representative, original FACS data are depicted as histograms and contour plots (n=3).

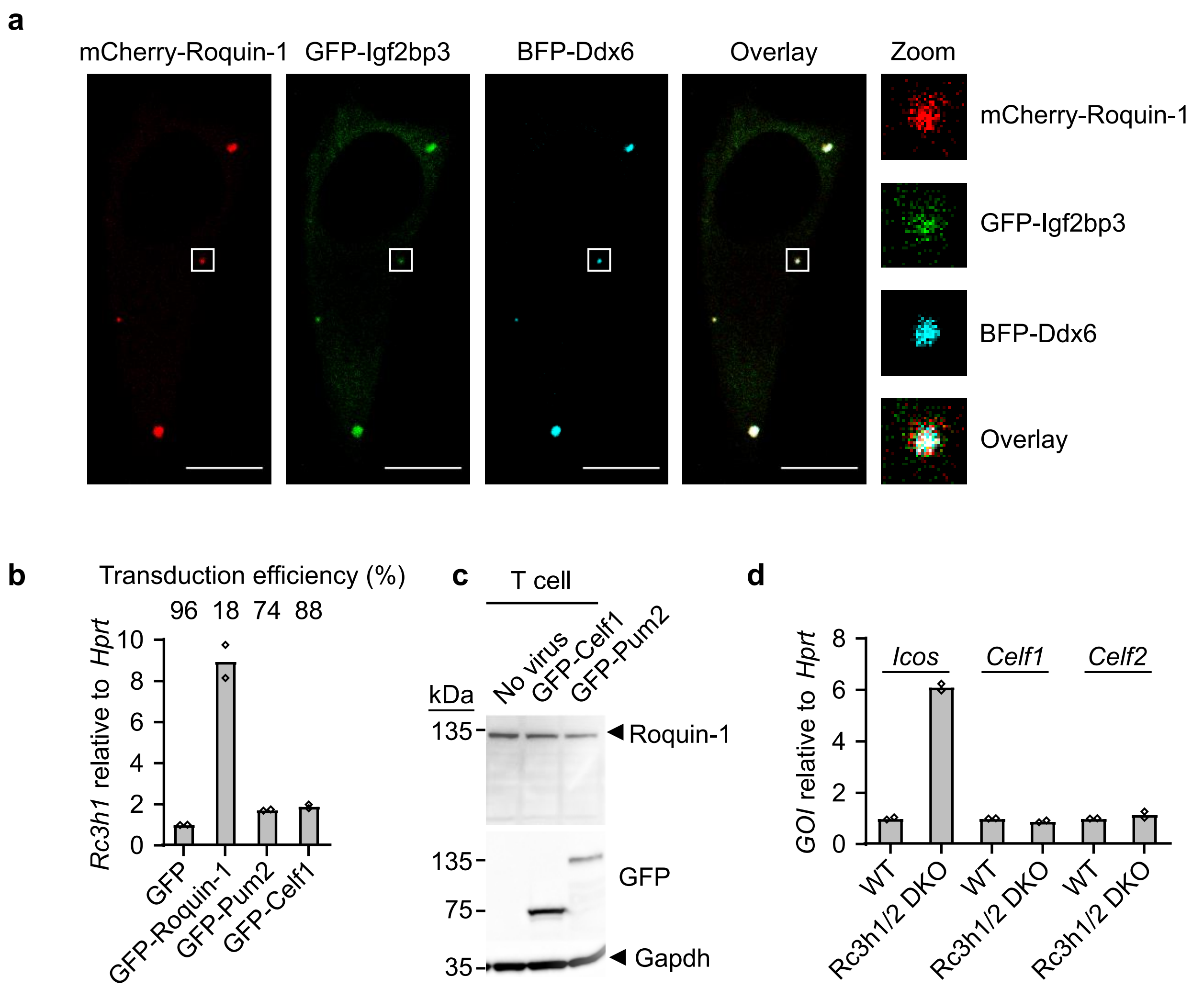
a
mCherry-Roquin-1
GFP-Igf2bp3
BFP-Ddx6
Overlay
Zoom
mCherry-Roquin-1
GFP-Igf2bp3
BFP-Ddx6
Overlay
b
Transduction efficiency (%)
96 18 74 88
Rc3h1 relative to Hprt
10
8
6
4
2
0
GFP
GFP-Roquin-1
GFP-Pum2
GFP-Celf1
c
T cell
No virus
GFP-Celf1
GFP-Pum2
kDa
135
Roquin-1
135
75
GFP
35
Gapdh
d
GOI relative to Hprt
8
6
4
2
0
Icos
Celf1
Celf2
WT
Rc3h1/2 DKO
WT
Rc3h1/2 DKO
WT
Rc3h1/2 DKO

Suppl. Fig. 8

Supplementary Fig. 8: Supporting results for higher order Icos regulation by Roquin-1 and Celf1 or Igf2bp3. (**a**) Using confocal microscopy, co-localization of Roquin-1 and Igf2bp3 in P bodies was demonstrated by co-transfecting Hela cells using plasmids coding for Cherry-Roquin-1, GFP-Igf2bp3 and BFP-Ddx6, the latter of which served as a P body marker (n=2). The standard bars equal 10 µm. (**b, c**) Cells taken from a ‘T cell-induced expression’ experiment as depicted in **Suppl. Fig. 7a**. (**b**) qPCR (n=1) and (**c**) Western blot (n=1) performed on cDNA or protein lysates, respectively, derived from $CD4^+$ T cells (WT) after doxycycline-induced expression of the indicated fusion proteins. (**d**) Quantitative PCR analysis of *Icos*, *Celf1*, and *Celf2* mRNA levels in WT and Roquin-1/2 DKO $CD4^+$ T cells (n=1). For (**b**) and (**d**) the data points represent technical replicates from qPCR results.

a

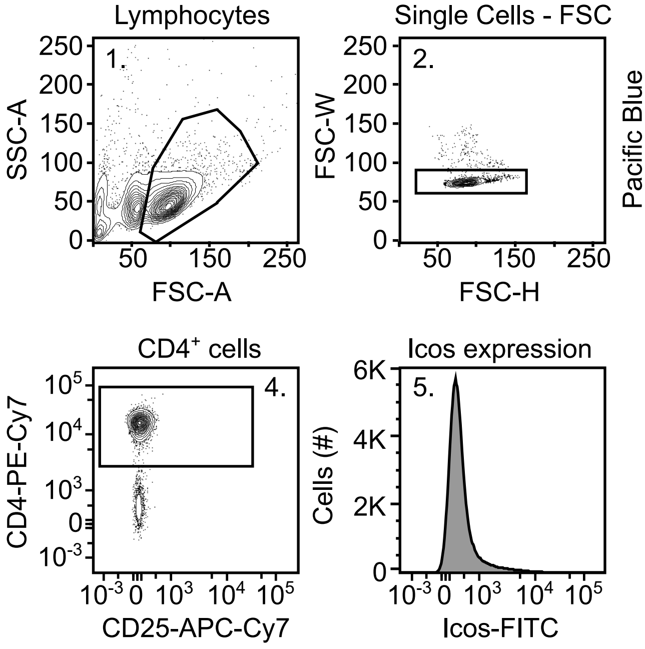

b

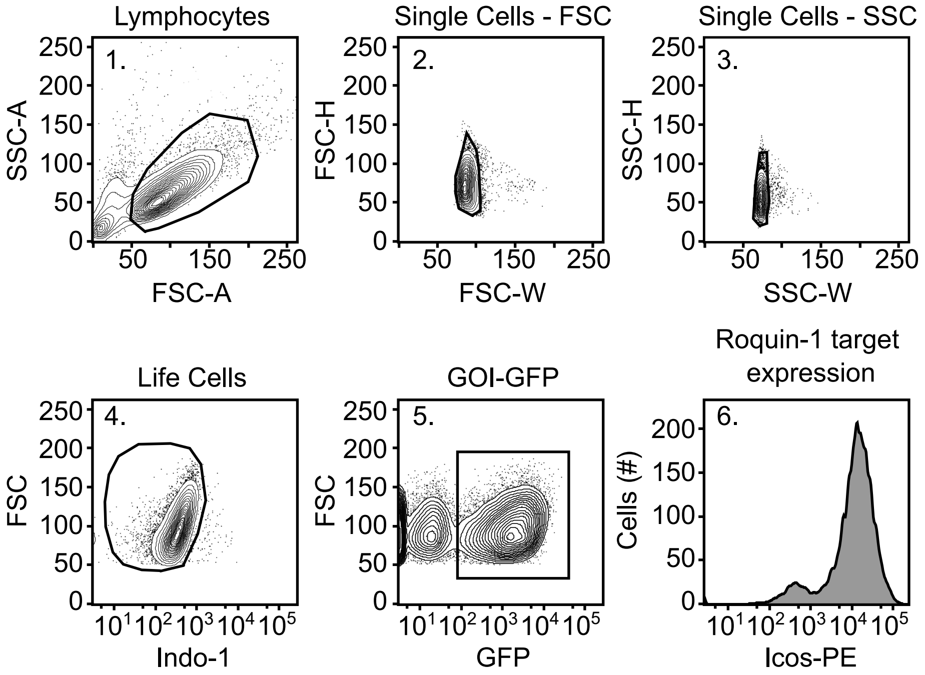

Suppl. Fig. 9

Supplementary Fig. 9: Gating strategies. (**a**) Depicts the gating strategy used for measuring Icos expression in Tamoxifen-induced KO and WT CD4$^+$ T cells, as used in **Fig. 1**. (**b**) Depicts the gating strategy for measuring Roquin-1/2 target gene expression after induced GOI-GFP expression in WT or 4'-OH-tamixifen-deleted Roquin-1/2 CD4$^+$ T cells (used in **Fig. 7** and **Suppl. Fig. 7**).

Supplementary table 1:

Proteins with annotated zf-CCCH domains according to the EuRBPDB database

Mus musculus		Homo sapiens	
RNA-IC	OOPS	RNA-IC	OOPS
Cpsf4	Cpsf4	CPSF4	CPSF4
Cpsf4l	Cpsf4l	CPSF4L	CPSF4L
Dhx57	Dhx57	DHX57	DHX57
Helz	Helz	HELZ	HELZ
Leng9	Leng9		
Mbnl1	Mbnl1	MBNL1	MBNL1
Mbnl2	Mbnl2	MBNL2	MBNL2
Mbnl3	Mbnl3	MBNL3	MBNL3
Mkrn1	Mkrn1	MKRN1	MKRN1
Mkrn2	Mkrn2	MKRN2	MKRN2
Mkrn3	Mkrn3		
Parp12	Parp12	PARP12	PARP12
Ppp1r10	Ppp1r10	PPP1R10	PPP1R10
Prr3	Prr3	PRR3	PRR3
Rbm27	Rbm27	RBM27	RBM27
Rc3h1	Rc3h1	RC3H1	RC3H1
Rc3h2	Rc3h2	RC3H2	RC3H2
Rnf113a1	Rnf113a1	RNF113A	RNF113A
Rnf113a2	Rnf113a2	RNF113B	RNF113B
Toe1	Toe1	TOE1	TOE1
Trmt1	Trmt1	TRMT1	TRMT1
U2af1	U2af1	U2AF1	U2AF1
U2af1l4	U2af1l4	U2AF1L4	U2AF1L4
		U2AF1L5	U2AF1L5
Unk	Unk	UNK	UNK
Unkl	Unkl	UNKL	UNKL
Zc3h10	Zc3h10	ZC3H10	ZC3H10
Zc3h13	Zc3h13	ZC3H13	ZC3H13
Zc3h15	Zc3h15	ZC3H15	ZC3H15
		ZC3H18	ZC3H18
Zc3h3	Zc3h3	ZC3H3	ZC3H3
Zc3h4	Zc3h4	ZC3H4	ZC3H4
Zc3h6	Zc3h6	ZC3H6	ZC3H6
		ZC3H7A	ZC3H7A
Zc3h7b	Zc3h7b	ZC3H7B	ZC3H7B
Zc3h8	Zc3h8	ZC3H8	ZC3H8
Zfp36	Zfp36	ZFP36	ZFP36
Zfp36l1	Zfp36l1	ZFP36L1	ZFP36L1
Zfp36l2	Zfp36l2	ZFP36L2	ZFP36L2
Zfp36l3	Zfp36l3		
Zmat5	Zmat5	ZMAT5	ZMAT5
Zrsr1	Zrsr1		
Zrsr2	Zrsr2	ZRSR2	ZRSR2

Highlighted in color are those canonical RBP’s that were identified by the respective method in the respective species.

Supplementary table 2:

Proteins with annotated KH domains according to the EuRBPDB database

Mus musculus		Homo sapiens	
RNA-IC	OOPS	RNA-IC	OOPS
4921511C20Rik	4921511C20Rik		
Akap1	Akap1	AKAP1	AKAP1
Ankhd1	Ankhd1	ANKHD1	ANKHD1
		ANKHD1-EIF4EBP3	ANKHD1-EIF4EBP3
Ankrd17	Ankrd17	ANKRD17	ANKRD17
Ascc1	Ascc1	ASCC1	ASCC1
Bicc1	Bicc1	BICC1	BICC1
Ddx43	Ddx43	DDX43	DDX43
		DDX53	DDX53
Fmr1	Fmr1	FMR1	FMR1
Fubp1	Fubp1	FUBP1	FUBP1
Fubp3	Fubp3	FUBP3	FUBP3
Fxr1	Fxr1	FXR1	FXR1
Fxr2	Fxr2	FXR2	FXR2
Gm382	Gm382		
Hdlbp	Hdlbp	HDLBP	HDLBP
Hnrnpk	Hnrnpk	HNRNPK	HNRNPK
Igf2bp1	Igf2bp1	IGF2BP1	IGF2BP1
Igf2bp2	Igf2bp2	IGF2BP2	IGF2BP2
Igf2bp3	Igf2bp3	IGF2BP3	IGF2BP3
Khdrbs1	Khdrbs1	KHDRBS1	KHDRBS1
Khdrbs2	Khdrbs2	KHDRBS2	KHDRBS2
Khdrbs3	Khdrbs3	KHDRBS3	KHDRBS3
Khsrp	Khsrp	KHSRP	KHSRP
Mex3a	Mex3a	MEX3A	MEX3A
Mex3b	Mex3b	MEX3B	MEX3B
Mex3c	Mex3c	MEX3C	MEX3C
Mex3d	Mex3d	MEX3D	MEX3D
Nova1	Nova1	NOVA1	NOVA1
Nova2	Nova2	NOVA2	NOVA2
Pcbp1	Pcbp1	PCBP1	PCBP1
Pcbp2	Pcbp2	PCBP2	PCBP2
Pcbp3	Pcbp3	PCBP3	PCBP3
Pcbp4	Pcbp4	PCBP4	PCBP4
Pnpt1	Pnpt1	PNPT1	PNPT1
Qk	Qk	QKI	QKI
Sf1	Sf1		
Tdrkh	Tdrkh	TDRKH	TDRKH

Highlighted in color are those canonical RBP’s that were identified by the respective method in the respective species.

Supplementary table 3:

Roquin-1 BioID candidates from T cells overlapping with the CD4⁺ T cell RBPome

1	Abcf1	Fnbp1	Prrc2c	Tnrc6b
2	Alyref	Helz	Pspc1	Ubap2
3	Atxn2l	Hist1h1c	R3hdm2	Ubap2l
4	Cnot1	Hist1h3b	Rc3h1	Upf1
5	Cnot2	Lsm14a	Rpl23a	Xrn1
6	Ddx6	Nufip2	Smg7	Ybx1
7	Ebna1bp2	Pc	Ssb	Ythdf1
8	Edc4	Plec	Stat3	Ythdf2
9	Eif4enif1	Prrc2a	Tdrd3	
10	Fam120a	Prrc2b	Tnrc6a	

Highlighted in blue are genes that were cloned for induced expression

Supplementary table 4:

Roquin-1 BioID candidates from MEF cells overlapping with $CD4^{+}$ T cell RBPome

1	Acaca	Fam120a	Noc3l	R3hdm2	Tdrd3
2	Ankrd17	Fmr1	Nop16	Rbms1	Tnpo1
3	Aqr	Fubp1	Nop56	Rbms2	Tnrc6a
4	Atxn2	Fxr1	Nufip2	Rc3h1	Tnrc6b
5	Atxn2l	Fxr2	Nup98	Riok1	Tnrc6c
6	Caprin1	G3bp1	Otud4	Rpl23a	Ubap2
7	Celf1	Gigyf2	Pabpc1	Rpl26	Ubap2l
8	Celf2	Gnb2l1	Patl1	Rpl27a	Upf1
9	Cenpe	Gnl3	Pc	Rpl6	Xrn1
10	Cnot1	Hist1h1c	Pds5b	Rpl8	Ybx1
11	Cnot11	Igf2bp3	Picalm	Rps14	Ybx3
12	Cnot2	Kdm3b	Plec	Rps26	Ythdf1
13	Cnot3	Kif1c	Prdx4	Rsl1d1	Ythdf2
14	Cpeb4	Larp4	Prrc2a	Smg7	Ythdf3
15	Csde1	Larp4b	Prrc2b	Snw1	Zc3hav1
16	Ddx18	Lsm14a	Prrc2c	Stat4	Zcchc6
17	Ddx27	Lsm14b	Pspc1	Stau1	
18	Ddx6	Lyar	Ptbp1	Strap	
19	Dhx9	Marf1	Pum1	Syncrip	
20	Eif4enif1	Mbnl2	Pum2	Tardbp	

Highlighted in color are genes that were cloned for induced expression. Blue: Detected in T and MEF cell BioIDs. Red: Detected in MEF cell BioIDs only.

Supplementary table 5: Primers

Convential cloning			
Gene name	Restriction Enzyme	Forward Primer	Reverse Primer
Crip1	*Hind*III/ *Kpn*I	AAGCTTGGGGGTACCATGCC GAAGTGCCC	CTACTTGAAAGTGTGGCTCTC AGCTCCACCTCGCC
Ldha	*Hind*III/ *Kpn*I	AAGCTTGGGGGTACCATGGC AACCCTCAA	TTAGAACTGCAGCTCCTTCTG GATTCCCCAGAGG
Rbms1	*Hind*III/ *Kpn*I	AAGCTTGGGGGTACCATGAT CTTCCCCAG	TTACTTATTGGGTGGAAAGGT ATATGGAGAATGGTCATTAGA CG
Stat1	*Hind*III/ *Kpn*I	AAGCTTGGGGGTACCATGTC ACAGTGGTT	TTATACTGTGCTCATCATACT GTCAAATTCGGGGCC
Stat4	*Hind*III/ *Kpn*I	AAGCTTGGGGGTACCATGTC TCAGTGGAA	TCATTCAGCAGAATATGGGG AATTCATTGCAGTTTCA
quantitative PCR			
Gene name		Forward Primer	Reverse Primer
Hprt		TCAGTCAACGGGGGACATAA A	GGGGCTGTACTGCTTAACCA G
β-actin		GGCTGTATTCCCCTCCATCG	CCAGTTGGTAACAATGCCAT GT
18S rRNA		GGCTGTATTCCCCTCCATCG	CCATCCAATCGGTAGTAGCG
Probe-based quantitative PCR			
Gene name	UPL Probe (#)	Forward Primer	Reverse Primer
Rc3h1	22	GAGACAGCACCTTACCAGCA	GACAAAGCGGGACACACAT
Icos	33	AACCTTAGTGGAGGATATTTG CAT	CTACGGGTAGCCAGTAGCTT C
Ctla4	21	TCACTGCTGTTTCTTTGAGCA	GGCTGAAATTGCTTTTCACAT
Nfkbid	21	ACTTCTCCCCTCCTCTGGTC	TCCGGAATCCACAGTCTCTT
Celf1	11	AGCAAGGCAGCAGCTGAG	CTTGTCTGATCCACAAATATA CACAG
Celf2	5	CCCTCTGTCTAGGACAAGCA	TCCCAAGAGAGGTCAAGGAA
Hprt	95	TCCTCCTCAGACCGCTTTT	CCTGGTTCATCATCGCTAATC
Retro-X-tight and pGEX backbone amplification			
Gene name		Forward Primer	Reverse Primer
RXT		GCGGCCGCACTCGAGATATC	GCCGCCTGAGCCGCCTGAG CCGCCCTTGTACAGCTCGTC CATGCC
pGEX-6P-2		GCGGCCGCATCGTGACTGAC TGACG	GGGAATTCCTGGGGATCCC
GOI sequencing			
		Forward Primer	Reverse Primer
RXT insert		CGACCACTACCAGCAGAACA	GATATCTCGAGACGCCGATG

PCR amplification and Infusion cloning of target genes into RXT			
gene name		Forward Primer	Reverse Primer
Eif4enif1		GGCGGCTCAGGCGGCGAGAAAAGTGTGGCTGAAACAGA	CTCGAGTGCGGCCGCTCACTGTCTATATTCCAGTTCATCT
Abcf1		GGCGGCTCAGGCGGCCCGAAGGGTCCCAAGCAAC	CTCGAGTGCGGCCGCTCAATCCCGAGGACGGTTGAC
Aqr		GGCGGCTCAGGCGGCGCGGCTCCTGCGCAGCCCAAGAAAA	CTCGAGTGCGGCCGCCTACTCGGTCTCTGTGGGGACA
Caprin1		GGCGGCTCAGGCGGCCCCTCGGCCACCAGCCAC	CTCGAGTGCGGCCGCTTAATTCACTTGCTGAGTGTTCA
Celf1		GGCGGCTCAGGCGGCGCTGCGTTTAAGTTGGATTTCC	CTCGAGTGCGGCCGCTCAGTAGGGCTTACTATCATTCTTC
Cnot2		GGCGGCTCAGGCGGCGTGAGGACTGATGGACATACA	CTCGAGTGCGGCCGCTTAGAAGGCTTGCTGAGCAGGG
Cpeb4		GGCGGCTCAGGCGGCGGGGATTACGGGTTTGGAGTG	CTCGAGTGCGGCCGCTCAGTTCCAGCGGAATGAAATATGC
Csde1		GGCGGCTCAGGCGGCAGCTTTGATCCAAACCTTCTCCAC	CTCGAGTGCGGCCGCTTAGTCAATGACACCAGCTTGAC
Ddx6		GGCGGCTCAGGCGGCAGCACGGCCAGAACAGAG	CTCGAGTGCGGCCGCTTACGGTTTCTCGTCTTCTGCAGG
Ebna1bp2		GGCGGCTCAGGCGGCGACACCCCTCCGCTTTCAGAG	CTCGAGTGCGGCCGCTCAGCGGGCTTTACTCTTCAG
Edc4		GGCGGCTCAGGCGGCGCCTCCTGCGCGAGCATC	CTCGAGTGCGGCCGCCTAAGGGAGGCTAGGGGTCAC
Fam120a		GGCGGCTCAGGCGGCGGCGTGCAGGGCTTCCAG	CTCGAGTGCGGCCGCTTACTCTTCTTTATTTAAGACAGC
Fmr1		GGCGGCTCAGGCGGCGAGGAGCTGGTGGTGGAAG	CTCGAGTGCGGCCGCTTATTTAGGGTACTCCATTCACCAG
Fubp1		GGCGGCTCAGGCGGCGCCGACTACTCCACAGTGC	CTCGAGTGCGGCCGCTTATTGGCCCTGAGGTGCTGG
Fxr1		GGCGGCTCAGGCGGCGCGGAGCTGACGGTGGAG	CTCGAGTGCGGCCGCTTATGAAACACCATTCAGGACTGC
Fxr2		GGCGGCTCAGGCGGCGGCGGCCTGGCCTCTGGG	CTCGAGTGCGGCCGCTTATGAAACTCCATTCACCAAACT
G3bp1		GGCGGCTCAGGCGGCGTTATGGAGAAGCCTAGTCCCC	CTCGAGTGCGGCCGCTCACTGCCTTGGAGTTGTAATCCC
Gigyf2		GGCGGCTCAGGCGGCGCAGCAGAAACACAGACAC	CTCGAGTGCGGCCGCTCAGTAGTCATCCAGAGTCTCAATC
Igf2bp3		GGCGGCTCAGGCGGCAACAAATTGTACATCGGGAACCTCA	CTCGAGTGCGGCCGCTTACTTCCGCCTTGACTGAGGT
Larp4b		GGCGGCTCAGGCGGCACTTCTGATCAGGACGCTAAAG	CTCGAGTGCGGCCGCTCACTGAGGAGACTTGGGAGG
Lsm14a		GGCGGCTCAGGCGGCAGCGGGGGCACCCCTTAC	CTCGAGTGCGGCCGCTTAGGGTCCAAAAGCCGTGG
Marf1		GGCGGCTCAGGCGGCGAAGGGAAAGGAACTGAGAACCC	CTCGAGTGCGGCCGCTTAAAGCTTGGTTACAGGTGC
Mbnl2		GGCGGCTCAGGCGGCGCCTTGAACGTTGCCCCC	CTCGAGTGCGGCCGCTTATTTCAGAATTATCTGATTGGC
Nufip2		GGCGGCTCAGGCGGCGAGGAGAAGCCCGGCCAG	CTCGAGTGCGGCCGCTCATTGATCTGGACTATCCATGGC
Patl1		GGCGGCTCAGGCGGCTTCCGCTACGAGTCTTTGGAGG	CTCGAGTGCGGCCGCTTACCGTATCCCCTGAACCAGC

Ptbp1		GGCGGCTCAGGCGGCGACG GCATCGTCCCAGACA	CTCGAGTGCGGCCGCCTAGA TGGTGGACTTGGAAAAGGA
Pum1		GGCGGCTCAGGCGGCAGCG TTGCATGTGTCTTGAAG	CTCGAGTGCGGCCGCTCAGA TGATACCATTAGGGGGACC
Pum2		GGCGGCTCAGGCGGCAATCA TGATTTTCAAGCTCTTGC	CTCGAGTGCGGCCGCTTACA GCATCCCATTTGGTG
R3hdm2		GGCGGCTCAGGCGGCTCTAA CAGTAACACTACTCAGGAG	CTCGAGTGCGGCCGCCTATT GAGAGCTAGCTCGTTCCAG
Rbms1		GGCGGCTCAGGCGGCATCTT CCCCAGCGGCAGC	CTCGAGTGCGGCCGCTTACT TATTGGGTGGAAAGG
Smg7		GGCGGCTCAGGCGGCAGGA CCGAAAACTTGAAATCAGAG G	CTCGAGTGCGGCCGCTCAGT GTGGAGGGTTCATGGC
Snw1		GGCGGCTCAGGCGGCGCGC TCACCAGCTTTTTACC	CTCGAGTGCGGCCGCCTACT CTTTCCTCCGCTTCTTGC
Stat3		GGCGGCTCAGGCGGCGCTC AGTGGAACCAGCTGC	CTCGAGTGCGGCCGCTCACA TGGGGGAGGTAGC
Stat4		GGCGGCTCAGGCGGCTCTCA GTGGAATCAAGTCCA	CTCGAGTGCGGCCGCTCATT CAGCAGAATATGGGGA
Stau		GGCGGCTCAGGCGGCTATAA GCCCGTGGACCCTCACTC	CTCGAGTGCGGCCGCTCAGC ACCTCCCGCACGC
Strap1		GGCGGCTCAGGCGGCGCCA TGAGGCAGACGCCG	CTCGAGTGCGGCCGCTCAGG CCTTAACTTCAGGAGTTG
Syncrip		GGCGGCTCAGGCGGCGCTA CAGAACATGTTAATGGAAATG	CTCGAGTGCGGCCGCCTACT TCCACTGTTGCCCAAAAG
Tardbp3		GGCGGCTCAGGCGGCTCTG AATATATTCGGGTAACAGAAG	CTCGAGTGCGGCCGCCTACA TTCCCCAGCCAGAAGAC
Tnpo1		GGCGGCTCAGGCGGCGTGT GGGACCGGCAAACC	CTCGAGTGCGGCCGCTTAAA CACCATAAAAAGCTGCAAGA
Ubap2		GGCGGCTCAGGCGGCATGA CTTCTGTGAGCAATGATCGT	CTCGAGTGCGGCCGCCTAGT TTGTCCAGTATGGAGCGCT
Ybx1		GGCGGCTCAGGCGGCAGCA GCGAGGCCGAGACC	CTCGAGTGCGGCCGCTTACT CAGCCCCGCCCTGC
Ythdf1		GGCGGCTCAGGCGGCTCGG CCACCAGCGTGGAC	CTCGAGTGCGGCCGCTTATT GTTTGTTTCGATTCTGTC
Ythdf2		GGCGGCTCAGGCGGCTCGG CCAGCAGCCTCTTG	CTCGAGTGCGGCCGCCTATT TCCCACGACCTTGACGTTC
Ythdf3		GGCGGCTCAGGCGGCTCAG CCACTAGCGTGGATCAG	CTCGAGTGCGGCCGCTTATT GCTTGTTTCTATTTCTCTCC
Zc3hav1		GGCGGCTCAGGCGGCACGG ATCCCGAGGTATTCTGTTTC	CTCGAGTGCGGCCGCTTATT TTCTCTGAAGGCCACTGGAG
Xrn1		GGCGGCTCAGGCGGCGGAG TCCCCAAGTTTTACCGATG	CTCGAGTGCGGCCGCTTATT CCGAAGGTTTAGAAACGCTG
PCR amplification and Infusion cloning of target genes into pGEX-6P-2			
Gene name		Forward Primer	Reverse Primer
mStat1		TCCCCAGGAATTCCCATGTC ACAGTGGTTCGAGC	TCACGATGCGGCCGCTTATA CTGTGCTCATCATACTGTC
mStat4		TCCCCAGGAATTCCCATGTCT CAGTGGAATCAAGTCC	TCACGATGCGGCCGCTCATT CAGCAGAATATGGGGAATTC
hStat1a		TCCCCAGGAATTCCCATGTCT CAGTGGTACGAACTTCAG	TCACGATGCGGCCGCCTATA CTGTGTTCATCATACTGTCG
hStat1b		TCCCCAGGAATTCCCATGTCT CAGTGGTACGAACTTCAG	TCACGATGCGGCCGCTTACA CTTCAGACACAGAAATCAAC

hStat4		TCCCCAGGAATTCCCATGTCTCAGTGGAATCAAGTCC	TCACGATGCGGCCGCTCATTCAGCAGAATAAGGAGACTTC

Sequences in red indicate overlap with plasmid backbone for infusion cloning
