# Supplementary material for: Defining the RBPome of primary T helper cells to elucidate higher-order Roquin-mediated mRNA regulation
Source: Nat Commun. 2021 Sep 1;12:5208. doi: 10.1038/s41467-021-25345-5 (PMC8410761; doi:10.1038/s41467-021-25345-5)
Supplement: Supplementary file 3 — Description of Additional Supplementary Files [file 41467_2021_25345_MOESM3_ESM.pdf]

Description of Additional Supplementary Files

File Name: Supplementary Data 1
Description: **Mass spectrometry data used to identify true-positive RNA-binding proteins in RNA-IC and OOPS experiments.** RNA-IC data: LFQ intensities after imputation are shown. In the T-test Significant column all significant right-sided outliers at a FDR of 5% are highlighted with a '+'. Proteins with a '+' in the column 'Significant by unique ID' were determined to be RNA-binding proteins by showing at least two identifications in the crosslinked sample and no identifications in the non-crosslinked sample. p-value, q-value, statistic and enrichment calculated by the two-sided student's t-tests comparing the crosslinked and the non-crosslinked sample are reported. Proteins identified as significant hits when enabling matching between runs are shown for Th0 cells. OOPS data: Same as for RNA-IC data. RNA-binding proteins were only determined by Student's T-test significance. The organic phase after RNAse treatment of the interphase of the crosslinked sample was compared to the same phase of the non-crosslinked sample to identify RBPs. Original data before imputation and filtering is shown in the "Output" data sheets.

File Name: Supplementary Data 2
Description: **Mass spectrometry data used to identify Roquin-1 preys in BioID experiments in T cells and in MEF cells.** Each protein was quantified based on summed unique peptide intensities and normalisation to total peptide intensities to compensate for loading differences. Ratios were calculated based on mean accumulated peptide intensities per proteins and a protein was defined as a Roquin-1 prey if it was identified by more than one unique peptide, had a p-value below 0.05 as calculated by student´s T-Test and was enriched as compared to BirA* controls by >1.99 fold (indicated by (+) in column H)
