# Supplementary material for: Defining the RBPome of primary T helper cells to elucidate higher-order Roquin-mediated mRNA regulation
Source: Nat Commun. 2021 Sep 1;12:5208. doi: 10.1038/s41467-021-25345-5 (PMC8410761; doi:10.1038/s41467-021-25345-5)
Supplement: Supplementary file 6 — Reporting Summary [file 41467_2021_25345_MOESM6_ESM.pdf]

nature research

Corresponding author(s): Vigo Heissmeyer

Last updated by author(s): Nov 30, 2020

Reporting Summary

Nature Research wishes to improve the reproducibility of the work that we publish. This form provides structure for consistency and transparency in reporting. For further information on Nature Research policies, see our Editorial Policies and the Editorial Policy Checklist.

Statistics

For all statistical analyses, confirm that the following items are present in the figure legend, table legend, main text, or Methods section.

n/a	Confirmed	
☐	☒	The exact sample size (*n*) for each experimental group/condition, given as a discrete number and unit of measurement
☐	☒	A statement on whether measurements were taken from distinct samples or whether the same sample was measured repeatedly
☐	☒	The statistical test(s) used AND whether they are one- or two-sided<br>*Only common tests should be described solely by name; describe more complex techniques in the Methods section.*
☒	☐	A description of all covariates tested
☐	☒	A description of any assumptions or corrections, such as tests of normality and adjustment for multiple comparisons
☐	☒	A full description of the statistical parameters including central tendency (e.g. means) or other basic estimates (e.g. regression coefficient) AND variation (e.g. standard deviation) or associated estimates of uncertainty (e.g. confidence intervals)
☐	☒	For null hypothesis testing, the test statistic (e.g. F, t, r) with confidence intervals, effect sizes, degrees of freedom and P value noted<br>*Give P values as exact values whenever suitable.*
☒	☐	For Bayesian analysis, information on the choice of priors and Markov chain Monte Carlo settings
☒	☐	For hierarchical and complex designs, identification of the appropriate level for tests and full reporting of outcomes
☒	☐	Estimates of effect sizes (e.g. Cohen's *d*, Pearson's *r*), indicating how they were calculated

Our web collection on statistics for biologists contains articles on many of the points above.

Software and code

Policy information about availability of computer code

Data collection	RBPome: Thermo Q-Exactive HF Tune software (v2.4.0.1824), Thermo Q-Exactive HF-X Tune software (v2.9.0.2982), Xcalibur (v4.0.27.19)<br>BioID: Progenesis QI software (Nonlinear Dynamics, Waters), LC-MSMS on a QExactive HF mass spectrometer (ThermoFisher Scientific) couple to an Ultimate 3000 RSLC nano HPLC
Data analysis	RBPome: MaxQuant (v1.5.1.6/1.5.6.7), Perseus (v1.6.0.28), R (v4.1.0)<br>BioID: Mascot search engine (V2.5.1 and V2.6.1)<br>BD FACS_DIVA (V8.0.1)<br>FlowJo (V10.5.3)

For manuscripts utilizing custom algorithms or software that are central to the research but not yet described in published literature, software must be made available to editors and reviewers. We strongly encourage code deposition in a community repository (e.g. GitHub). See the Nature Research guidelines for submitting code & software for further information.

Data

Policy information about availability of data

All manuscripts must include a data availability statement. This statement should provide the following information, where applicable:

- Accession codes, unique identifiers, or web links for publicly available datasets
- A list of figures that have associated raw data
- A description of any restrictions on data availability

The mass spectrometry data that support the RBP identification by RNA-IC and OOPS in CD4 T cells have been deposited at PRIDE with the accession codes PXD022795, PXD021169, PXD021164, PXD008830.

The BioID data sets have also been deposited at PRIDE with the accession code PXD026716.

Field-specific reporting

Please select the one below that is the best fit for your research. If you are not sure, read the appropriate sections before making your selection.

- [x] Life sciences
- [ ] Behavioural & social sciences
- [ ] Ecological, evolutionary & environmental sciences

For a reference copy of the document with all sections, see nature.com/documents/nr-reporting-summary-flat.pdf

Life sciences study design

All studies must disclose on these points even when the disclosure is negative.

Sample size

Fig. 1: One mouse per genotype in the T cell activation time courses, three independent repeats (n=3).
Suppl. Fig. 1d: Comparison of UV irradiated vs non-irradiated samples in EL-4 cell (n=2)
Fig. 2: RNA-IC; TH0-differentiated CD4 T cells from one C57Bl/6 mouse or one human donor per sample (CL vs nCL; n=3)
Suppl. Fig 2: RNA-IC: iTreg-differentiated CD4 T cells from one C57Bl/6 mouse or one human donor per sample (CL vs nCL; n=3)
Fig. 3: OOPS; TH0-differentiated CD4 T cells from one C57Bl/6 mouse or one human donor per sample (CL vs nCL; n=4)
Suppl. Fig. 3c and 3d are based on the samples of Fig. 3 (n=4)
Fig. 5: In vitro binding assay (Fig. 5a: n= 3; Fig. 5b: n=2); tethering assays in triplicates, with two independent repeats (n=2)
Fig. 6: BirA-GFP vs BirA-Roquin-1 samples in T cells (n=5)
Suppl. Fig. 6: BirA vs BirA-Roquin-1 samples in MEF cells (n=4)
Fig. 7: Induced expression of GFP-GOI in Roquin iDKO or WT cells (n=3)
Sample sizes n=2 were used in situations were a method was established or when results from different methods supported each other.
Samples size n>3 were used whenever it was technically and economically feasible to exceed n=3.

Data exclusions

No data were excluded from this study.

Replication

All experiments were repeatable. The number of replications for each experiment is listed above (sample size).

Randomization

No randomization was performed. Variations in between inbred animals are small. Earliest available mice of the correct genotype and age were chosen for experiments. Human blood samples were obtained from volunteer fellow researchers. We did not perform population studies and overlap of results in molecular methods (OOPS/RNA-IC) was strong despite different ages and sexes of donors.

Blinding

The investigators were not blinded since the experiments were performed in an unbiased manner by mass spectrometers, FACS devices, computer programs and algorithms.

Reporting for specific materials, systems and methods

We require information from authors about some types of materials, experimental systems and methods used in many studies. Here, indicate whether each material, system or method listed is relevant to your study. If you are not sure if a list item applies to your research, read the appropriate section before selecting a response.

Materials & experimental systems

n/a	Involved in the study	
[ ]	[x]	Antibodies
[ ]	[x]	Eukaryotic cell lines
[x]	[ ]	Palaeontology and archaeology
[ ]	[x]	Animals and other organisms
[ ]	[x]	Human research participants
[x]	[ ]	Clinical data
[x]	[ ]	Dual use research of concern

Methods

n/a	Involved in the study	
[x]	[ ]	ChIP-seq
[ ]	[x]	Flow cytometry
[x]	[ ]	MRI-based neuroimaging

Antibodies

Antibodies used

All in-house monoclonal antibody supernatants generated at the Helmholtz Center were use in a dilution of 1:10 for FACS and Western blot applications. All commercial antibodies used for FACS were diluted 1:200.
Western blots and IPs: anti-Roquin-1/2, cl. 3F12 (in-house production); anti-Regnase-1, cl. 15D11 (in-house production); anti-pan-Ago, cl. MAGO3-5 (in-house production), anti-Nufip2, cl. 23G8 (in-house production), anti-Fxr1, polyclonal, 4173, 1:1000, (Cell Signaling); anti-Fxr2, cl. D85D6, 7098, 1:1000 (Cell Signaling); anti-TTP, cl. TP6, 1:1000 (Sigma); anti-pan-YTHDF, cl. 17F2 (in-house production); anti-Gapdh, cl. 6C5, CB1001, 1:10000 (Calbiochem), anti-Celf1, cl. 850717, MAB9388, 0.5 µg/ml (R&D SYSTEMS); anti-RBMS1, cl. EPR9825(B), ab150353, 1:5000 (abcam); anti-CPEB4, polyclonal, 25342-1-AP, 1:750 (proteintech); anti-GFP, cl. 3E5-111 (in-house production); anti-Ptbp1, 1:1000, 8776 (Cell Signaling); anti-tubulin, 1:1000, 86298 (Cell Signaling); goat anti-rat antibody, cl. Poly4054, 1:200 (Biolegend); goat anti-mouse antibody, polyclonal, 554001, 1:400 (BD Bioscience); rabbit anti-roquin, A300-514A, 6

µg/IP (Bethyl); mouse monoclonal anti-Celf1, ab9549, 2 µg/IP (Abcam).
T cell culture: anti-CD28, cl. 37.5N, 2.5 µg/ml (in-house production); anti-CD3, cl. 2C11, 0.5 µl/ml (in-house production); anti-IL-4, cl. 11B11, 10 µg/ml (in-house production); goat anti-hamster, 56984, 1:20 in PBS (MP Biochemicals).
FACS: anti-CD4, cl. GK1.5, 25-0041-82, 1:1000 (eBioscience); anti-CD44, cl. IM7, 1:200 (eBioscience), anti-CD62L, cl. MEL-14, 1:200 (eBioscience), anti-CD25, cl. PC61, 1:200 (Biolegend), anti-Icos-FITC, cl. 7E17G9, 11-9942-82 (eBioscience); anti-Wtap, cl. 4A10G9, 60188-1-Ig, 1:200 (Proteintech); anti-Dgcr8-APC, cl. EPR18757, ab221302, 1:100 (Abcam); anti-Icos-PE, cl. 7E.17G9, 12-9942-82 (eBioscience); anti-Ox40-APC, cl. OX-86, 17-1341-82 (eBioscience); anti-Ctla4-PE, UC10-4B9, 12-1522-81 (eBioscience); anti-IkBNS, cl. 4C1 rat monoclonal (in-house production); anti-Regnase-1, cl. 15D11 rat monoclonal (in-house production); anti-rat-AF647, cl. poly4054, 405416 (Biolegend).

Validation

In-house generated antibodies were tested in ELISAs and validated against overexpression and/or KO controls. Commercial antibodies were purchased only if the intended use was documented in previous publications and/or on the website of the company. The purchased antibodies were additionally validated by comparison of WT vs KO/knockdown or WT vs overexpression situations.

Eukaryotic cell lines

Policy information about cell lines

Cell line source(s)

HEK 293T, EL-4 and HeLa cells were obtained from the American Type Culture Collection (ATCC).
MEF were generated, immortalization, genetically manipulated and cloned in-house.

Authentication

The cells were not authenticated.

Mycoplasma contamination

All cell lines that were used in this publication were tested for mycoplasma and were found to be negative.

Commonly misidentified lines
(See ICLAC register)

No cell lines that have been reported as misidentified have been used in this study.

Animals and other organisms

Policy information about studies involving animals; ARRIVE guidelines recommended for reporting animal research

Laboratory animals

For CD4+ T cell isolation 6 to 10 week old male or female mice were used. Animals were housed under a 12h/12h dark/light regime at 20-24 °C and at a humidity of 45-65%. Conditional knockout mice Rc3h1-2fl/fl or Zc3h12afl/fl or Wtapfl/fl or Dgcr8fl/fl were crossed with CD4-Cre-ERT2 mice to generate C57Bl/6 mice of the respective genotypes:
1. Rc3h1-2fl/fl;CD4-Cre-ERT2
2. Zc3h12afl/fl;CD4-Cre-ERT2
3. Dgcr8fl/fl;CD4-Cre-ERT2
4. Wtapfl/fl;CD4-Cre-ERT2
Rc3h1-2fl/fl; CD4-Cre-ERT2 mice were crossed with rtTA transgenic mice to generate C57Bl/6 mice of the genotype:
5. Rc3h1-2fl/fl; CD4-Cre-ERT2; rtTA mice
Details are reported in Methods.

Wild animals

The study did not involve wild animals.

Field-collected samples

None.

Ethics oversight

Animal breeding and experimentation followed the legal approval of the Government of Upper Bavaria (Regierung von Oberbayern, reference numbers 55.2-2532-VET_02-19-122 and 55.2-2532.Vet_02-19-68). The work was compliant with the relevant ethical regulations for animal testing and research. All animals were housed in a pathogen-free barrier facility in accordance with the Helmholtz Zentrum München, the Ludwig-Maximilians-University München institutional, state, and federal guidelines.

Note that full information on the approval of the study protocol must also be provided in the manuscript.

Human research participants

Policy information about studies involving human research participants

Population characteristics

Blood was donated from healthy volunteering young science students and older scientists (Caucasian, 5 male, 2 female age: 27-58y). Donors had no history of autoimmune disease or recent illness. A significant influence of population characteristics on the identification of RNA-binding proteins by RNA-IC/OOPS and MS seems unlikely, but can not categorically be excluded.

Recruitment

Students and co-workers from the lab environment. To avoid any seletive bias, we included male and female adults of different age.

Ethics oversight

PBMC were purified using standard procedures from peripheral blood obtained from healthy volunteer donors after informed consent. The use of material of human origin in this work was approved by the ethics committee of the Technical University of Munich (approvals 934/03 and 1872/07) and was in accordance with the Declaration of Helsinki of the World Medical Association (last amended in 2013).

Note that full information on the approval of the study protocol must also be provided in the manuscript.

Flow Cytometry

Plots

Confirm that:

- [x] The axis labels state the marker and fluorochrome used (e.g. CD4-FITC).
- [x] The axis scales are clearly visible. Include numbers along axes only for bottom left plot of group (a 'group' is an analysis of identical markers).
- [x] All plots are contour plots with outliers or pseudocolor plots.
- [x] A numerical value for number of cells or percentage (with statistics) is provided.

Methodology

Sample preparation	CD4+ T cell isolation (STEMCELL), followed by 24h of 4'OH tamoxifen treatment, if applicable (induced GFP-GOI expression, Fig. 7). Anti-CD3 (2C11) and anti-CD28 (37.5N) activation of CD4+ T cells followed on goat anti-hamster IgG-coated plates (MP Biochemicals) for 48h without skewing antibodies (RNA-IC and OOPS; Fig. 2-4) or with Th1 skewing antibodies in time-course (Fig. 1) or BioID (Fig. 6) experiments (including anti-IL-4 (cl. 11B11), IL-12 (BD Pharmingen). Viral transduction was performed after 40h of activation, if applicable (induced GFP-GOI expression; Fig. 7). After 48h of activation, CD4+ T cells were resuspended and expanded in IL-2- containing medium for two days. If applicable, doxycycline-induced expression was initiated 16h prior to analysis (BirA-Roquin; Fig. 6/ GFP-GOI; Fig. 7). Unfixed cells were stained with Icos-PE (clone 7E.17G9) and Ox40-APC (clone OX-86). Intracellular staining of formaldehyde-fixed, saponin-permeabilized cells was performed separately for Ctla4-PE (UC10-4B9) and IkBNS (4C1) plus anti-rat-AF647 antibody (cl. poly4054) and for Regnase-1 (15D11, rat monoclonal), plus anti-rat-AF647 antibody (cl. poly4054).
Instrument	LSRII, LSRFortessa (BD Biosciences), FACS Canto II (3-laser)
Software	FlowJo_V10 (V10.5.3), BD FACSDiva (V8.0.1)
Cell population abundance	Isolated CD4+ T cells were typically >95% positive for the CD4 surface marker.
Gating strategy	From the lymphocyte gate (SSC-A/FSC-A) single cells (FSC_H vs FSC_W and SSC-H vs SSC-W) that were viable (fixable violet negative) were gated for GFP positivity, indicating doxycycline-induced expression of the transduced GFP fusion gene. Untransduced cells were used as a negative control and GFP-only transduced cells functioned as a positive control and as reference for GFP-GOI-induced effects on Roquin-1 targets. Target expression was displayed in histograms. A gating strategy is provided in Suppl. Figure 9.

- [x] Tick this box to confirm that a figure exemplifying the gating strategy is provided in the Supplementary Information.
