# Supplementary material for: Defining the RBPome of primary T helper cells to elucidate higher-order Roquin-mediated mRNA regulation
Source: Nat Commun. 2021 Sep 1;12:5208. doi: 10.1038/s41467-021-25345-5 (PMC8410761; doi:10.1038/s41467-021-25345-5)
Supplement: Supplementary file 7 — Supplementary Tables [file 41467_2021_25345_MOESM7_ESM.pdf]

Supplementary table 1:

Proteins with annotated zf-CCCH domains according to the EuRBPDB database

Mus musculus		Homo sapiens	
RNA-IC	OOPS	RNA-IC	OOPS
Cpsf4	Cpsf4	CPSF4	CPSF4
Cpsf4l	Cpsf4l	CPSF4L	CPSF4L
Dhx57	Dhx57	DHX57	DHX57
Helz	Helz	HELZ	HELZ
Leng9	Leng9		
Mbnl1	Mbnl1	MBNL1	MBNL1
Mbnl2	Mbnl2	MBNL2	MBNL2
Mbnl3	Mbnl3	MBNL3	MBNL3
Mkrn1	Mkrn1	MKRN1	MKRN1
Mkrn2	Mkrn2	MKRN2	MKRN2
Mkrn3	Mkrn3		
Parp12	Parp12	PARP12	PARP12
Ppp1r10	Ppp1r10	PPP1R10	PPP1R10
Prr3	Prr3	PRR3	PRR3
Rbm27	Rbm27	RBM27	RBM27
Rc3h1	Rc3h1	RC3H1	RC3H1
Rc3h2	Rc3h2	RC3H2	RC3H2
Rnf113a1	Rnf113a1	RNF113A	RNF113A
Rnf113a2	Rnf113a2	RNF113B	RNF113B
Toe1	Toe1	TOE1	TOE1
Trmt1	Trmt1	TRMT1	TRMT1
U2af1	U2af1	U2AF1	U2AF1
U2af1l4	U2af1l4	U2AF1L4	U2AF1L4
		U2AF1L5	U2AF1L5
Unk	Unk	UNK	UNK
Unkl	Unkl	UNKL	UNKL
Zc3h10	Zc3h10	ZC3H10	ZC3H10
Zc3h13	Zc3h13	ZC3H13	ZC3H13
Zc3h15	Zc3h15	ZC3H15	ZC3H15
		ZC3H18	ZC3H18
Zc3h3	Zc3h3	ZC3H3	ZC3H3
Zc3h4	Zc3h4	ZC3H4	ZC3H4
Zc3h6	Zc3h6	ZC3H6	ZC3H6
		ZC3H7A	ZC3H7A
Zc3h7b	Zc3h7b	ZC3H7B	ZC3H7B
Zc3h8	Zc3h8	ZC3H8	ZC3H8
Zfp36	Zfp36	ZFP36	ZFP36
Zfp36l1	Zfp36l1	ZFP36L1	ZFP36L1
Zfp36l2	Zfp36l2	ZFP36L2	ZFP36L2
Zfp36l3	Zfp36l3		
Zmat5	Zmat5	ZMAT5	ZMAT5
Zrsr1	Zrsr1		
Zrsr2	Zrsr2	ZRSR2	ZRSR2

Highlighted in color are those canonical RBP’s that were identified by the respective method in the respective species.

Supplementary table 2:

Proteins with annotated KH domains according to the EuRBPDB database

Mus musculus		Homo sapiens	
RNA-IC	OOPS	RNA-IC	OOPS
4921511C20Rik	4921511C20Rik		
Akap1	Akap1	AKAP1	AKAP1
Ankhd1	Ankhd1	ANKHD1	ANKHD1
		ANKHD1-EIF4EBP3	ANKHD1-EIF4EBP3
Ankrd17	Ankrd17	ANKRD17	ANKRD17
Ascc1	Ascc1	ASCC1	ASCC1
Bicc1	Bicc1	BICC1	BICC1
Ddx43	Ddx43	DDX43	DDX43
		DDX53	DDX53
Fmr1	Fmr1	FMR1	FMR1
Fubp1	Fubp1	FUBP1	FUBP1
Fubp3	Fubp3	FUBP3	FUBP3
Fxr1	Fxr1	FXR1	FXR1
Fxr2	Fxr2	FXR2	FXR2
Gm382	Gm382		
Hdlbp	Hdlbp	HDLBP	HDLBP
Hnrnpk	Hnrnpk	HNRNPK	HNRNPK
Igf2bp1	Igf2bp1	IGF2BP1	IGF2BP1
Igf2bp2	Igf2bp2	IGF2BP2	IGF2BP2
Igf2bp3	Igf2bp3	IGF2BP3	IGF2BP3
Khdrbs1	Khdrbs1	KHDRBS1	KHDRBS1
Khdrbs2	Khdrbs2	KHDRBS2	KHDRBS2
Khdrbs3	Khdrbs3	KHDRBS3	KHDRBS3
Khsrp	Khsrp	KHSRP	KHSRP
Mex3a	Mex3a	MEX3A	MEX3A
Mex3b	Mex3b	MEX3B	MEX3B
Mex3c	Mex3c	MEX3C	MEX3C
Mex3d	Mex3d	MEX3D	MEX3D
Nova1	Nova1	NOVA1	NOVA1
Nova2	Nova2	NOVA2	NOVA2
Pcbp1	Pcbp1	PCBP1	PCBP1
Pcbp2	Pcbp2	PCBP2	PCBP2
Pcbp3	Pcbp3	PCBP3	PCBP3
Pcbp4	Pcbp4	PCBP4	PCBP4
Pnpt1	Pnpt1	PNPT1	PNPT1
Qk	Qk	QKI	QKI
Sf1	Sf1		
Tdrkh	Tdrkh	TDRKH	TDRKH

Highlighted in color are those canonical RBP’s that were identified by the respective method in the respective species.

Supplementary table 3:

Roquin-1 BioID candidates from T cells overlapping with the CD4$^+$ T cell RBPome

1	Abcf1	Fnbp1	Prrc2c	Tnrc6b
2	Alyref	Helz	Pspc1	Ubap2
3	Atxn2l	Hist1h1c	R3hdm2	Ubap2l
4	Cnot1	Hist1h3b	Rc3h1	Upf1
5	Cnot2	Lsm14a	Rpl23a	Xrn1
6	Ddx6	Nufip2	Smg7	Ybx1
7	Ebna1bp2	Pc	Ssb	Ythdf1
8	Edc4	Plec	Stat3	Ythdf2
9	Eif4enif1	Prrc2a	Tdrd3	
10	Fam120a	Prrc2b	Tnrc6a	

Highlighted in blue are genes that were cloned for induced expression

Supplementary table 4:

Roquin-1 BioID candidates from MEF cells overlapping with CD4$^+$ T cell RBPome

1	Acaca	Fam120a	Noc3l	R3hdm2	Tdrd3
2	Ankrd17	Fmr1	Nop16	Rbms1	Tnpo1
3	Aqr	Fubp1	Nop56	Rbms2	Tnrc6a
4	Atxn2	Fxr1	Nufip2	Rc3h1	Tnrc6b
5	Atxn2l	Fxr2	Nup98	Riok1	Tnrc6c
6	Caprin1	G3bp1	Otud4	Rpl23a	Ubap2
7	Celf1	Gigyf2	Pabpc1	Rpl26	Ubap2l
8	Celf2	Gnb2l1	Patl1	Rpl27a	Upf1
9	Cenpe	Gnl3	Pc	Rpl6	Xrn1
10	Cnot1	Hist1h1c	Pds5b	Rpl8	Ybx1
11	Cnot11	Igf2bp3	Picalm	Rps14	Ybx3
12	Cnot2	Kdm3b	Plec	Rps26	Ythdf1
13	Cnot3	Kif1c	Prdx4	Rsl1d1	Ythdf2
14	Cpeb4	Larp4	Prrc2a	Smg7	Ythdf3
15	Csde1	Larp4b	Prrc2b	Snw1	Zc3hav1
16	Ddx18	Lsm14a	Prrc2c	Stat4	Zcchc6
17	Ddx27	Lsm14b	Pspc1	Stau1	
18	Ddx6	Lyar	Ptbp1	Strap	
19	Dhx9	Marf1	Pum1	Syncrip	
20	Eif4enif1	Mbnl2	Pum2	Tardbp	

Highlighted in color are genes that were cloned for induced expression. Blue: Detected in T and MEF cell BioIDs. Red: Detected in MEF cell BioIDs only.

Supplementary table 5: Primers

Convential cloning			
Gene name	Restric-tion Enzyme	Forward Primer	Reverse Primer
Crip1	*Hind*III/ *Kpn*I	AAGCTTGGGGGTACCATGCCGAAGTGCCC	CTACTTGAAAGTGTGGCTCTCAGCTCCACCTCGCC
Ldha	*Hind*III/ *Kpn*I	AAGCTTGGGGGTACCATGGCAACCCTCAA	TTAGAACTGCAGCTCCTTCTGGATTCCCCAGAGG
Rbms1	*Hind*III/ *Kpn*I	AAGCTTGGGGGTACCATGATCTTCCCCAG	TTACTTATTGGGTGGAAAGGTATATGGAGAATGGTCATTAGACG
Stat1	*Hind*III/ *Kpn*I	AAGCTTGGGGGTACCATGTCACAGTGGTT	TTATACTGTGCTCATCATACTGTCAAATTCGGGGCC
Stat4	*Hind*III/ *Kpn*I	AAGCTTGGGGGTACCATGTCTCAGTGGAA	TCATTCAGCAGAATATGGGGAATTCATTGCAGTTTCA
quantitative PCR			
Gene name		Forward Primer	Reverse Primer
Hprt		TCAGTCAACGGGGGACATAAA	GGGGCTGTACTGCTTAACCAG
β-actin		GGCTGTATTCCCCTCCATCG	CCAGTTGGTAACAATGCCATGT
18S rRNA		GGCTGTATTCCCCTCCATCG	CCATCCAATCGGTAGTAGCG
Probe-based quantitative PCR			
Gene name	UPL Probe (#)	Forward Primer	Reverse Primer
Rc3h1	22	GAGACAGCACCTTACCAGCA	GACAAAGCGGGACACACAT
Icos	33	AACCTTAGTGGAGGATATTTGCAT	CTACGGGTAGCCAGTAGCTTC
Ctla4	21	TCACTGCTGTTTCTTTGAGCA	GGCTGAAATTGCTTTTCACAT
Nfkbid	21	ACTTCTCCCCTCCTCTGGTC	TCCGGAATCCACAGTCTCTT
Celf1	11	AGCAAGGCAGCAGCTGAG	CTTGTCTGATCCACAAATATACACAG
Celf2	5	CCCTCTGTCTAGGACAAGCA	TCCCAAGAGAGGTCAAGGAA
Hprt	95	TCCTCCTCAGACCGCTTTT	CCTGGTTCATCATCGCTAATC
Retro-X-tight and pGEX backbone amplification			
Gene name		Forward Primer	Reverse Primer
RXT		GCGGCCGCACTCGAGATATC	GCCGCCTGAGCCGCCTGAGCCGCCCTTGTACAGCTCGTCCATGCC
pGEX-6P-2		GCGGCCGCATCGTGACTGACTGACG	GGGAATTCCTGGGGATCCC
GOI sequencing			
		Forward Primer	Reverse Primer
RXT insert		CGACCACTACCAGCAGAACA	GATATCTCGAGACGCCGATG

PCR amplification and Infusion cloning of target genes into RXT			
gene name		Forward Primer	Reverse Primer
Eif4enif1		GGCGGCTCAGGCGGCGAGA AAAGTGTGGCTGAAACAGA	CTCGAGTGCGGCCGCTCACT GTCTATATTCCAGTTCATCT
Abcf1		GGCGGCTCAGGCGGCCCGA AGGGTCCCAAGCAAC	CTCGAGTGCGGCCGCTCAAT CCCGAGGACGGTTGAC
Aqr		GGCGGCTCAGGCGGCGCGG CTCCTGCGCAGCCCAAGAAA A	CTCGAGTGCGGCCGCCTACT CGGTCTCTGTGGGGACA
Caprin1		GGCGGCTCAGGCGGCCCCT CGGCCACCAGCCAC	CTCGAGTGCGGCCGCTTAAT TCACTTGCTGAGTGTTCA
Celf1		GGCGGCTCAGGCGGCGCTG CGTTTAAGTTGGATTTCC	CTCGAGTGCGGCCGCTCAGT AGGGCTTACTATCATTCTTC
Cnot2		GGCGGCTCAGGCGGCGTGA GGACTGATGGACATACA	CTCGAGTGCGGCCGCTTAGA AGGCTTGCTGAGCAGGG
Cpeb4		GGCGGCTCAGGCGGCGGGG ATTACGGGTTTGGAGTG	CTCGAGTGCGGCCGCTCAGT TCCAGCGGAATGAAATATGC
Csde1		GGCGGCTCAGGCGGCAGCT TTGATCCAAACCTTCTCCAC	CTCGAGTGCGGCCGCTTAGT CAATGACACCAGCTTGAC
Ddx6		GGCGGCTCAGGCGGCAGCA CGGCCAGAACAGAG	CTCGAGTGCGGCCGCTTACG GTTTCTCGTCTTCTGCAGG
Ebna1bp2		GGCGGCTCAGGCGGCGACA CCCCTCCGCTTTCAGAG	CTCGAGTGCGGCCGCTCAGC GGGCTTTACTCTTCAG
Edc4		GGCGGCTCAGGCGGCGCCT CCTGCGCGAGCATC	CTCGAGTGCGGCCGCCTAAG GGAGGCTAGGGGTCAC
Fam120a		GGCGGCTCAGGCGGCGGCG TGCAGGGCTTCCAG	CTCGAGTGCGGCCGCTTACT CTTCTTTATTTAAGACAGC
Fmr1		GGCGGCTCAGGCGGCGAGG AGCTGGTGGTGGAAG	CTCGAGTGCGGCCGCTTATT TAGGGTACTCCATTCACCAG
Fubp1		GGCGGCTCAGGCGGCGCCG ACTACTCCACAGTGC	CTCGAGTGCGGCCGCTTATT GGCCCTGAGGTGCTGG
Fxr1		GGCGGCTCAGGCGGCGCGG AGCTGACGGTGGAG	CTCGAGTGCGGCCGCTTATG AAACACCATTCAGGACTGC
Fxr2		GGCGGCTCAGGCGGCGGCG GCCTGGCCTCTGGG	CTCGAGTGCGGCCGCTTATG AAACTCCATTCACCAAACT
G3bp1		GGCGGCTCAGGCGGCGTTAT GGAGAAGCCTAGTCCCC	CTCGAGTGCGGCCGCTCACT GCCTTGGAGTTGTAATCCC
Gigyf2		GGCGGCTCAGGCGGCGCAG CAGAAACACAGACAC	CTCGAGTGCGGCCGCTCAGT AGTCATCCAGAGTCTCAATC
Igf2bp3		GGCGGCTCAGGCGGCAACA AATTGTACATCGGGAACCTCA	CTCGAGTGCGGCCGCTTACT TCCGCCTTGACTGAGGT
Larp4b		GGCGGCTCAGGCGGCACTTC TGATCAGGACGCTAAAG	CTCGAGTGCGGCCGCTCACT GAGGAGACTTGGGAGG
Lsm14a		GGCGGCTCAGGCGGCAGCG GGGGCACCCCTTAC	CTCGAGTGCGGCCGCTTAGG GTCCAAAAGCCGTGG
Marf1		GGCGGCTCAGGCGGCGAAG GGAAAGGAACTGAGAACCC	CTCGAGTGCGGCCGCTTAAA GCTTGGTTACAGGTGC
Mbnl2		GGCGGCTCAGGCGGCGCCT TGAACGTTGCCCCC	CTCGAGTGCGGCCGCTTATT TCAGAATTATCTGATTGGC
Nufip2		GGCGGCTCAGGCGGCGAGG AGAAGCCCGGCCAG	CTCGAGTGCGGCCGCTCATT GATCTGGACTATCCATGGC
Patl1		GGCGGCTCAGGCGGCTTCC GCTACGAGTCTTTGGAGG	CTCGAGTGCGGCCGCTTACC GTATCCCCTGAACCAGC

Ptbp1		GGCGGCTCAGGCGGCGACG GCATCGTCCCAGACA	CTCGAGTGCGGCCGCCTAGA TGGTGGACTTGGAAAAGGA
Pum1		GGCGGCTCAGGCGGCAGCG TTGCATGTGTCTTGAAG	CTCGAGTGCGGCCGCTCAGA TGATACCATTAGGGGGACC
Pum2		GGCGGCTCAGGCGGCAATCA TGATTTTCAAGCTCTTGC	CTCGAGTGCGGCCGCTTACA GCATCCCATTTGGTG
R3hdm2		GGCGGCTCAGGCGGCTCTAA CAGTAACACTACTCAGGAG	CTCGAGTGCGGCCGCCTATT GAGAGCTAGCTCGTTCCAG
Rbms1		GGCGGCTCAGGCGGCATCTT CCCCAGCGGCAGC	CTCGAGTGCGGCCGCTTACT TATTGGGTGGAAAGG
Smg7		GGCGGCTCAGGCGGCAGGA CCGAAAACTTGAAATCAGAG G	CTCGAGTGCGGCCGCTCAGT GTGGAGGGTTCATGGC
Snw1		GGCGGCTCAGGCGGCGCGC TCACCAGCTTTTTACC	CTCGAGTGCGGCCGCCTACT CTTTCCTCCGCTTCTTGC
Stat3		GGCGGCTCAGGCGGCGCTC AGTGGAACCAGCTGC	CTCGAGTGCGGCCGCTCACA TGGGGGAGGTAGC
Stat4		GGCGGCTCAGGCGGCTCTCA GTGGAATCAAGTCCA	CTCGAGTGCGGCCGCTCATT CAGCAGAATATGGGGA
Stau		GGCGGCTCAGGCGGCTATAA GCCCGTGGACCCTCACTC	CTCGAGTGCGGCCGCTCAGC ACCTCCCGCACGC
Strap1		GGCGGCTCAGGCGGCGCCA TGAGGCAGACGCCG	CTCGAGTGCGGCCGCTCAGG CCTTAACTTCAGGAGTTG
Syncrip		GGCGGCTCAGGCGGCGCTA CAGAACATGTTAATGGAAATG	CTCGAGTGCGGCCGCCTACT TCCACTGTTGCCCAAAAG
Tardbp3		GGCGGCTCAGGCGGCTCTG AATATATTCGGGTAACAGAAG	CTCGAGTGCGGCCGCCTACA TTCCCCAGCCAGAAGAC
Tnpo1		GGCGGCTCAGGCGGCGTGT GGGACCGGCAAACC	CTCGAGTGCGGCCGCTTAAA CACCATAAAAAGCTGCAAGA
Ubap2		GGCGGCTCAGGCGGCATGA CTTCTGTGAGCAATGATCGT	CTCGAGTGCGGCCGCCTAGT TTGTCCAGTATGGAGCGCT
Ybx1		GGCGGCTCAGGCGGCAGCA GCGAGGCCGAGACC	CTCGAGTGCGGCCGCTTACT CAGCCCCGCCCTGC
Ythdf1		GGCGGCTCAGGCGGCTCGG CCACCAGCGTGGAC	CTCGAGTGCGGCCGCTTATT GTTTGTTTCGATTCTGTC
Ythdf2		GGCGGCTCAGGCGGCTCGG CCAGCAGCCTCTTG	CTCGAGTGCGGCCGCCTATT TCCCACGACCTTGACGTTC
Ythdf3		GGCGGCTCAGGCGGCTCAG CCACTAGCGTGGATCAG	CTCGAGTGCGGCCGCTTATT GCTTGTTTCTATTTCTCTCC
Zc3hav1		GGCGGCTCAGGCGGCACGG ATCCCGAGGTATTCTGTTTC	CTCGAGTGCGGCCGCTTATT TTCTCTGAAGGCCACTGGAG
Xrn1		GGCGGCTCAGGCGGCGGAG TCCCCAAGTTTTACCGATG	CTCGAGTGCGGCCGCTTATT CCGAAGGTTTAGAAACGCTG
PCR amplification and Infusion cloning of target genes into pGEX-6P-2			
Gene name		Forward Primer	Reverse Primer
mStat1		TCCCCAGGAATTCCCATGTC ACAGTGGTTCGAGC	TCACGATGCGGCCGCTTATA CTGTGCTCATCATACTGTC
mStat4		TCCCCAGGAATTCCCATGTCT CAGTGGAATCAAGTCC	TCACGATGCGGCCGCTCATT CAGCAGAATATGGGGAATTC
hStat1a		TCCCCAGGAATTCCCATGTCT CAGTGGTACGAACTTCAG	TCACGATGCGGCCGCCTATA CTGTGTTCATCATACTGTCG
hStat1b		TCCCCAGGAATTCCCATGTCT CAGTGGTACGAACTTCAG	TCACGATGCGGCCGCTTACA CTTCAGACACAGAAATCAAC

hStat4		TCCCCAGGAATTCCCATGTCTCAGTGGAATCAAGTCC	TCACGATGCGGCCGCTCATTCAGCAGAATAAGGAGACTTC

Sequences in red indicate overlap with plasmid backbone for infusion cloning
